# Quantitative multiplexed proteomics of *Taenia solium* cysts obtained from the skeletal muscle and central nervous system of pigs

**DOI:** 10.1371/journal.pntd.0005962

**Published:** 2017-09-25

**Authors:** José Navarrete-Perea, Marta Isasa, Joao A. Paulo, Ricardo Corral-Corral, Jeanette Flores-Bautista, Beatriz Hernández-Téllez, Raúl J. Bobes, Gladis Fragoso, Edda Sciutto, Xavier Soberón, Steven P. Gygi, Juan P. Laclette

**Affiliations:** 1 Dept. of Immunology, Institute for Biomedical Research, Universidad Nacional Autónoma de México, Ciudad de México, México; 2 Dept. of Cell Biology, Harvard Medical School, Boston, Massachusetts, United States of America; 3 Dept. of Biochemistry and Structural Biology, Institute of Cell Physiology, Universidad Nacional Autónoma de México, Ciudad de México, México; 4 Dept. of Tissue and Cell Biology, School of Medicine, Universidad Nacional Autónoma de México, Ciudad de México, México; 5 Instituto Nacional de Medicina Genómica, Ciudad de México, México; 6 Dept. of Biocatalysis and Cellular Engineering, Instituto de Biotecnología, Universidad Nacional Autónoma de México, Morelos, México; University of Würzburg, GERMANY

## Abstract

In human and porcine cysticercosis caused by the tapeworm *Taenia solium*, the larval stage (cysts) can infest several tissues including the central nervous system (CNS) and the skeletal muscles (SM). The cyst’s proteomics changes associated with the tissue localization in the host tissues have been poorly studied. Quantitative multiplexed proteomics has the power to evaluate global proteome changes in response to different conditions. Here, using a TMT-multiplexed strategy we identified and quantified over 4,200 proteins in cysts obtained from the SM and CNS of pigs, of which 891 were host proteins. To our knowledge, this is the most extensive intermixing of host and parasite proteins reported for tapeworm infections.Several antigens in cysticercosis, *i*.*e*., GP50, paramyosin and a calcium-binding protein were enriched in skeletal muscle cysts. Our results suggested the occurrence of tissue-enriched antigen that could be useful in the improvement of the immunodiagnosis for cysticercosis. Using several algorithms for epitope detection, we selected 42 highly antigenic proteins enriched for each tissue localization of the cysts. Taking into account the fold changes and the antigen/epitope contents, we selected 10 proteins and produced synthetic peptides from the best epitopes. Nine peptides were recognized by serum antibodies of cysticercotic pigs, suggesting that those peptides are antigens. Mixtures of peptides derived from SM and CNS cysts yielded better results than mixtures of peptides derived from a single tissue location, however the identification of the ‘optimal’ tissue-enriched antigens remains to be discovered. Through machine learning technologies, we determined that a reliable immunodiagnostic test for porcine cysticercosis required at least five different antigenic determinants.

## Author summary

Human and porcine cysticercosis caused by *Taenia solium* is a parasite disease still endemic in developing countries. The cysts can be located in different host tissues, including different organs of the central nervous system and the skeletal muscles. The molecular mechanisms associated with the tissue localization of the cysts are not well understood. Here, we described the proteome changes of the cysts obtained from different host tissues from infected pigs using quantitative multiplex proteomics. We explored the diversity of host proteins identified in the cyst’s protein extracts and we also explored the immune-localization of several host-related proteins within the cysts, and propose their possible function. We identified several proteins and antigens enriched for a given tissue localization. Several synthetic peptides designed from these tissue-enriched antigens were tested trough ELISA. Using a combination of peptide mixtures and machine learning technologies we were able to distinguish non cysticercotic and cysticercotic pig’s sera. The tissue-enriched proteins/antigens could be useful for the development of improved immuno-diagnostic tests capable of discriminate the tissue-localization of the cysts.

## Introduction

Human and porcine cysticercosis caused by the larval stage of *Taenia solium*, is acquired by the ingestion of this parasite’s eggs. After activation by several gastrointestinal agents, the oncospheres penetrating the intestinal wall later establish in different tissues and organs including the skeletal muscles (SM) and the brain. In humans, establishment of cysts in the central nervous system (CNS) causes neurocysticercosis (NC), a serious and pleomorphic disease that can become highly debilitating [1]. Heterogeneity of human NC has been associated, at least in part, with the number and localization of the cysts in the CNS [2], as well as to many other factors including a complex immune response directed to a number of cyst’s antigens [3, 4, 5, 6].

The molecular factors associated with the tissue localization of the *T*. *solium* cysts remain poorly understood [7]. Other pathogenic microorganisms (*S*. *pneumoniae*, *Campylobacter jejuni*, *Escherichia coli*, *Trypanosoma brucei*, etc.), show tissue preference linked to a number of specific pathogen’s proteins [8, 9, 10, 11, 12].

Information available on proteomics changes of flatworm parasite infections is limited. However, we know that parasites respond to hormones, cytokines and other host’s molecules [13]. The availability of several tapeworm genomes [14] has allowed to detail this complex host-parasite cross-communication including insulin, EGF/FGF, TGF-b/BMP, among others (for an updated review see [15]). Insulin responsiveness has been described for *Schistosoma mansoni*, *Taenia crassiceps* and *Echinococcus multilocularis* [16, 17, 18]. The differential effects of steroid hormones during parasite infections is also well documented [19]. Some parasites also have the ability to respond to host cytokines; for example, *S*. *mansoni* has receptors to TNF-α and TGF-β and proteomic and genomic changes have been reported in response to those cytokines [20, 21, 22].

The advent of high throughput proteomic techniques greatly widens our power to approach these old questions in molecular helminthology. In this context, body fluids of the host may affect proteome expression of infectious agents, for example, *E*. *coli* growing in a media supplemented with urine show a differential proteome signature [23]. Furthermore, several proteomic changes of *Streptococcus pyogenes* have been reported in response to serum supplementation [24]. The molecular factors associated with the tissue localization of helminth parasites within the host tissues has been less explored; in the case of *Trichinella spiralis* several changes have also been reported between parasites isolated from different host tissues [25]. However, important advances in helminth proteomics, including metacestode cystic/vesicular larval forms, have been reported [26–29].

It is conceivable that the host tissue’s molecular environment modulates the protein expression of pathogens, including parasites. Accordingly, specific proteomic profiles of parasites could be associated with a certain tissue localization.

Understanding the proteome changes of parasites in different host tissues, can provide insights not only on the molecular networking occurring in complex host parasite relationships, but it could also be useful for the design of more effective vaccines, drugs, as well as for the improvement of available diagnostic procedures.

Here we benefited from isobaric quantitative proteomics to elucidate the proteomic changes of *T*. *solium* cysts obtained of SM and CNS of pigs. A protein profile was found associated with each tissue localization, allowing the identification of 42 tissue-enriched antigens and the design of 14 synthetic antigenic peptides that were evaluated for antibody recognition using infected and uninfected pig’s sera. Our results indicated that an optimal immunological diagnosis for porcine cysticercosis requires at least five different epitopes from several tissue-enriched antigens. A remarkable finding was the conspicuous and abundant presence of host proteins in the protein extracts of the cysts; 891 host proteins were identified and quantified. We present initial findings suggesting that several intact host’s proteins might play a significant role in tapeworm’s physiology.

## Materials and methods

### Protein extracts

#### Cyst’s isolation and total protein extracts for quantitative proteomics

Cysticercotic pigs were humanely sacrificed by certified veterinary doctors in accordance with institutional protocols from the Institute for Biomedical Research and the School of Veterinary Medicine and Zootechnics at the Universidad Nacional Autónoma de México. *T*. *solium* cysts were dissected immediately after sacrifice from the skeletal muscle and the central nervous system of three experimentally and two naturally infected pigs, using only cysts in the vesicular stage, all the cysts included in this study had a similar appearance (size ≈ 1 cm, transparent bladder wall and crystalline vesicular fluid). The cysts were obtained under aseptic conditions, inflammatory capsules surrounding the cysts were carefully and completely removed to reduce the host contaminant material (except those bound to the cyst's surface), the larvae were washed several times with ice cold PBS, pH 7.3. Time elapsed between the sacrifice and the cyst’s isolation was no longer than 90 min. From each tissue and pig, five whole cysts from each animal and tissue were homogenized in 8M urea in 50 mM HEPES pH 8.0, complemented with protease inhibitors (Complete, Roche) using a polytron homogenizer. The extracts were centrifuged 15 min at 14,000 x g and the supernatants were collected. Afterwards, all supernatants were reduced (10 mM DTT for 1 h) and then alkylated (15 mM iodoacetamide) for 30 min in the dark; excess iodoacetamide was quenched using 15 mM DTT. A graphic explanation is provided in S1 Fig.

#### Antigenic extracts for ELISA and western blotting assays

The insoluble fraction of cysts tissue was obtained as described previously [30]. The vesicular fluid (VF) was obtained from skeletal muscle cysts from naturally infected pigs; the bladder wall was sectioned using a scalpel and the released VF from several cysts was pooled and diluted 1:2 using 50 mM Tris pH 7.3, complemented with protease inhibitors.

#### Total saline extracts for IgG purification

Cysts were obtained from the skeletal muscle of naturally infected pigs (n = 3), homogenized in PBS, pH 7.3, supplemented with 0.45 M of NaCl and protease inhibitors using a Polytron and centrifuged at 14,000 rpm for 15 min.

### Protein digestion and TMT labelling

Methanol−chloroform precipitation of the reduced and alkylated protein extracts was performed prior to protease digestion. Samples of 400 μg of each protein extract were resuspended separately in 100 μL of 8 M urea in 50 mM HEPES, pH 8.2. After solubilization, the protein extracts were diluted to 4 M urea with 50 mM HEPES, pH 8.2, and digested at RT for 3 h with endoproteinase Lys-C (Wako, Japan) at 5 ng/μL. The mixtures were then diluted to 1 M urea with 50 mM HEPES, pH 8.2, and trypsin was added at a 50:1 protein-to-protease ratio. The reaction was incubated overnight at 37°C and stopped by the addition of 100% TFA to a final pH < 2. Peptides were desalted using 50 mg tC18 SepPak solid-phase extraction cartridges (Waters, Milford, MA) and lyophilized. Desalted peptides were resuspended in 100 μL of 200 mM HEPES, pH 8.2. Peptide concentrations were determined using the microBCA assay (Thermo Fisher Scientific, Waltham, MA). One-hundred micrograms of peptides from each sample was labeled with TMT reagent. TMT-10 reagents (0.8 mg, from Thermo Fisher Scientific) were dissolved in anhydrous acetonitrile (40 μL), of which 10 μL were added to the peptides along with 30 μL of acetonitrile (final acetonitrile concentration of approximately 30% (v/v)). The labeling reaction proceeded for 1 h at room temperature and then was quenched with hydroxylamine (Sigma, St. Louis, MO) to a final concentration of 0.3% (v/v). The TMT-labeled samples were mixed equally, vacuum centrifuged to near dryness, desalted using 200 mg solid-phase C18 extraction cartridge (Sep-Pak, Waters), and lyophilized.

### Off-line Basic pH Reversed-Phase (BPRP) fractionation

The TMT-labeled peptides were fractionated using BPRP HPLC. An Agilent 1100 pump equipped with a degasser and a photodiode array (PDA) detector (set at 220 and 280 nm wavelength) from Thermo Fisher Scientific (Waltham, MA) were used. Peptides were subjected to a 50 min linear gradient from 5% to 35% acetonitrile in 10 mM ammonium bicarbonate pH 8 at a flow rate of 0.8 mL/min over an Agilent 300 Extend C18 column (5 μm particles, 4.6 mm ID, and 220 mm in length). Beginning at 10 min of peptide elution, fractions were collected every 0.38 min into a total of 96 fractions, which were consolidated into 24, of which 12 nonadjacent samples were analyzed. Samples were dried via vacuum centrifugation. Each eluted fraction was acidified with 1% formic acid and desalted using StageTips [31], dried via vacuum centrifugation, and reconstituted in 4% acetonitrile, 5% formic acid for LC−MS/MS analysis.

### Liquid chromatography and tandem mass spectrometry

All mass spectrometry data were collected on an Orbitrap Fusion mass spectrometer (Thermo Fisher Scientific, San Jose, CA) coupled to a Proxeon EASY-nLC II liquid chromatography (LC) pump (Thermo Fisher Scientific). Peptides were eluted over a 100 μm inner diameter micro-capillary column packed with ∼0.5 cm of Magic C4 resin (5 μm, 100 Å, Michrom Bioresources) followed by ∼35 cm of Accucore resin (2.6 μm, 150 Å, Thermo Fisher Scientific). For each analysis, we loaded ∼1 μg of the peptide mixture onto the column. Peptides were separated using a 90 min gradient of 6−26% acetonitrile in 0.125% formic acid at a flow rate of ∼350 nL/min. The dynamic exclusion duration was set at 90 s, with a mass tolerance of ±7 ppm. Each analysis used the multinotch MS3-based TMT method [32] on an Orbitrap Fusion mass spectrometer, which has been shown to reduce ion interference compared to MS2 quantification. The scan sequence began with an MS1 spectrum (Orbitrap analysis; resolution 120000; mass range 400−1400 m/z; automatic gain control (AGC) target 2 × 10^5^; maximum injection time 100 ms). The 10 most-abundant MS1 ions of charge states 2−6 were fragmented, and multiple MS2 ions were selected using a Top10 method. MS2 analysis was composed of collision induced dissociation (quadrupole ion trap analysis, AGC 4 × 10^3^; normalized collision energy (NCE) 35; maximum injection time 150 ms). Following acquisition of each MS2 spectrum, we collected an MS3 spectrum as described previously [32], in which multiple MS2 fragment ions were captured in the MS3 precursor population using isolation waveforms with multiple frequency notches. MS3 precursors were fragmented by high energy collision-induced dissociation (HCD) and analyzed using the Orbitrap (NCE 55; AGC 5 × 10^4^; maximum injection time 150 ms, resolution was 60,000 at 400 Th).

### Data analysis

Instrument data files were processed using a SEQUEST-based in-house software pipeline [33]. Spectra were converted from.raw to mzXML using a modified version of ReAdW.exe. A database containing all predicted ORFs for entries from the parasite (*T*. *solium* genome database; http://www.genedb.org/Homepage/Tsolium; downloaded March 31, 2015) and the host (*Sus scrofa* database; http://www.uniprot.org/proteomes/?query=taxonomy:9823; downloaded March 31, 2015) was used. This database was concatenated with another database composed of all protein sequences in the reverse order. Searches were performed using a 50 ppm precursor ion tolerance for total protein level analysis. The product ion tolerance was set to 0.9 Da. These wide mass tolerance windows were chosen to maximize sensitivity besides SEQUEST searches and linear discriminant analysis [34, 35]. TMT tags on lysine residues and peptide N termini (+229.163 Da) and carbamidomethylation of cysteine residues (+57.021 Da) were set as static modifications, while oxidation of methionine residues (+15.995 Da) was established as a variable modification.

**Fig 1:**
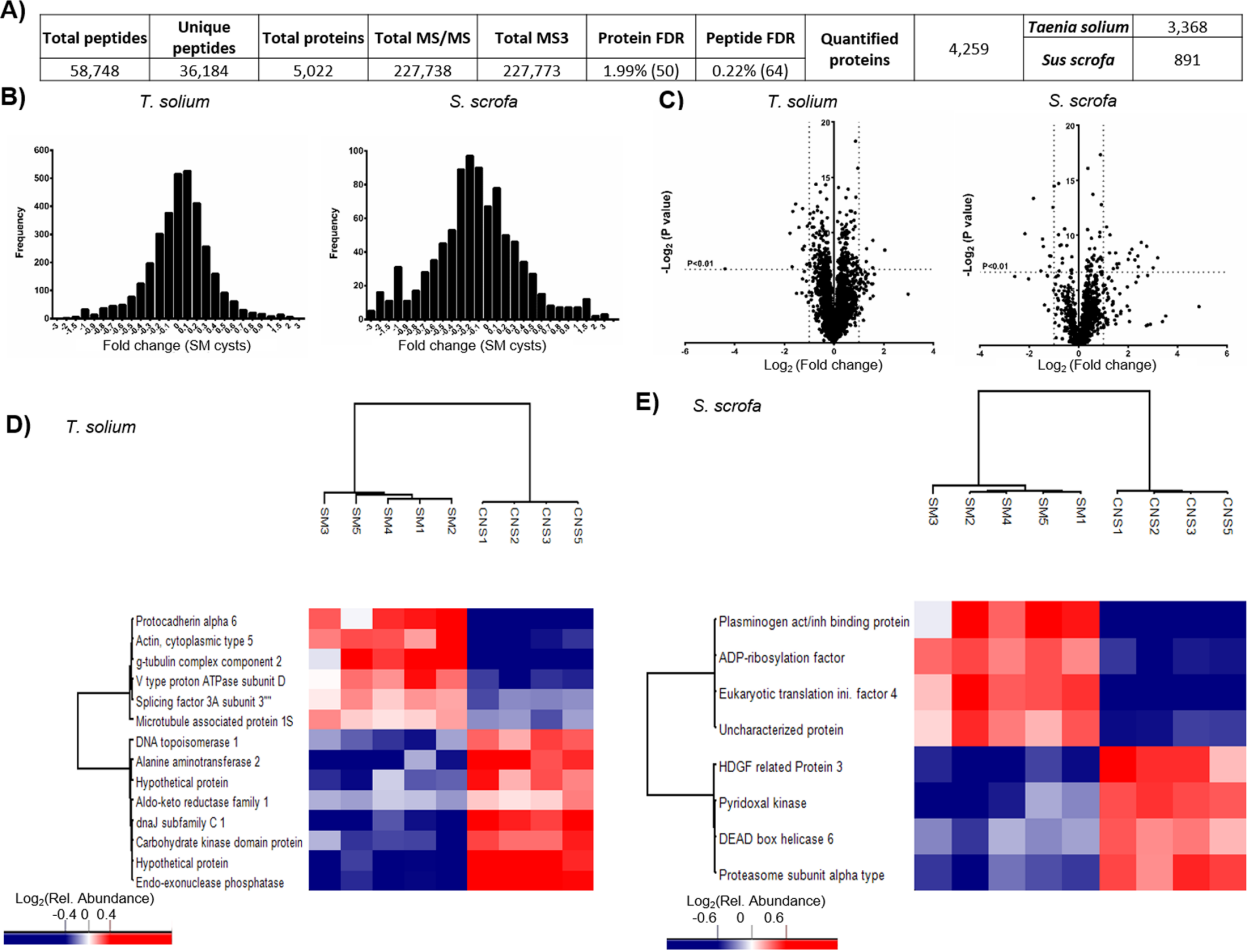
Proteomic analysis of *Taenia solium* cysts. A) Table summarizing peptide and protein quantification from the nine samples. Proteins were collapsed to a final protein-level FDR < 2%. B) Fold change distributions of proteins of skeletal muscle (SM) cysts. C) Volcano plots showing P-value and the Log_2_ (fold change CNS cysts/SM cysts) of proteins of SM and CNS cysts. D) Heat map showing the signature for *T*. *solium* proteins of SM and CNS cysts. E) Heat map showing the signature for the host (*Sus scrofa*) proteins quantified in protein extracts of SM and CNS cysts.

Peptide-spectrum matches (PSMs) were adjusted to a 2% false discovery rate (FDR) [35]. PSM filtering was performed using a linear discriminant analysis, as described previously [33], while considering the following parameters: XCorr, ΔCn, missed cleavages, peptide length, charge state, and precursor mass accuracy. For TMT-based reporter ion quantitation, we extracted the signal-to-noise (S/N) ratio for each TMT channel and found the closest matching centroid to the expected mass collapsed to a 1% peptide FDR and then collapsed further to a final protein-level FDR of 1%. Moreover, for protein assembly, principles of parsimony were used to produce the smallest protein set, necessary to account for all observed peptides. Proteins were quantified by summing reporter ion counts across all matching PSMs using in-house software, as described previously [36]. Briefly, a 0.003 Th window around the theoretical m/z of each reporter ion (126, 126.1278 Th; 127N, 127.1249 Th; 127C, 127.1310 Th; 128N, 128.1283 Th; 128C, 128.1343 Th; 129N, 129.1316 Th; 129C, 129.1377 Th; 130N, 130.1349 Th; 130C, 130.1410 Th; 131, 131.1382 Th) was scanned for ions, and the maximum intensity nearest the theoretical m/z was used. PSMs with poor quality, MS3 spectra with TMT reporter summed signal-to-noise ratio less than 387, or no MS3 spectra were excluded from quantitation [32]. The RAW files will be made available upon request. Protein quantitation values were exported for further analysis in Excel, Perseus 1.5.2.4 and GraphPad prism v6. Proteins with more than three missing channels were discarded, in the case of identifications based in a single peptide, that peptide was present in at least 7 samples. The selection of the tissue-enriched proteins (Fig 1) was based on the comparison of fold changes between CNS and SM cysts using a multiple T-test and Benjamini-Hochberg correction with a 5% of FDR (there were 5 CNS samples, unfortunately, one sample of CNS cysts was discarded at the end, due to poor data quality).

### Peptide selection for antibodies detection

Proteins with a P-value <0.01 (n = 261) and proteins without changes (lowest coefficient of variation, n = 50) were chosen to predict their antigenic regions. A detailed explanation is found in the S2 Fig. Initially, the antigenicity algorithm [37] and the B cell epitope algorithm [38] were used to quantitatively estimate the proteins with the higher antigenicity. Only the proteins predicted by both algorithms with a high percentage of antigen/epitope were selected (n = 42). The peptide selection was based on the following criteria: length of at least 15 amino acids (average size of predicted antigenic regions = 14.1); coincidence in the prediction of at least 5 amino acids by both algorithms, and at least, 10 amino acids should be predicted by one of the algorithm. The resulting peptides were submitted to an algorithm that was trained with a set of synthetic peptides of proven utility in diagnostic procedures as well as with a set of peptides that were not useful [39]; peptides with the highest probability of recognition by antibodies were selected. A total of ten peptides were selected (one from each protein); 4 peptides derived from proteins that were abundant in SM cysts, 4 from CNS cysts and 2 from proteins that did not show change in both tissues. All peptides were purchased from GenScript (USA).

### Protein sequence analysis

Proteins from the host (*Sus scrofa*) were annotated using the PantherGo algorithm [40, 41]; in the case of *T*. *solium* proteins, only proteins with a P-value<0.01 were submitted to Argot2 algorithm [42–44] using a threshold of 200. Disulfide bonds, N-linked glycosylation sites, transmembrane regions, signal peptides, and GPI-anchoring sites were predicted for selected proteins using several algorithms [45–56].

### Experimental validation of theoretically predicted antigenic peptides

The insoluble fraction of *T*. *solium* cysts, the VF and the synthetic peptides were tested by ELISA. Briefly, 1.5 μg of the insoluble fraction and of the VF, as well as 500 ng of each synthetic peptide were used to coat separate wells of microtiter plates. After overnight incubation at 4°C with mild agitation, the plates were washed, blocked for 2 h with 1% albumin in PBS-0.05% Tween 20 (PBST) and incubated with different pig sera, diluted 1:200 in PBST and incubated overnight at 4°C. A HRP coupled anti-pig IgG hyperimmune serum was used (diluted 1:4,000) as secondary antibody. The reaction was developed using OPD (0.4 mg/mL) for about 3–5 min and stopped with 3N HCl. Absorbance at 492 nm was determined in a Multiskan FC (Thermo-Fisher Scientific).

### Purification of pig IgG from a protein extract of *T*. *solium* cysts

The total saline extract was passed through a column of Protein G coupled to Sepharose 4B. The bound IgG was eluted using 0.1 M glycine pH 2.3 and immediately neutralized with Tris 1M, pH 7.3. Fractions containing the bound IgG were concentrated using an Amicon system (10 kDa cutoff) and washed several times using PBS, pH 7.3. The purified IgG was quantified by Non-Interfering protein assay (GBiosciences).

### Functional testing of the pig IgG purified from the cysts extracts

The IgG purified from the cysts protein extracts was tested for antibody activity through conventional ELISA and western-blotting procedures. For ELISA, microtiter plates were separately coated using 1.5 μg of VF or the insoluble protein fraction of cyst tissue (see above) in carbonate buffer, pH 9.6. After overnight incubation at 4°C with mild agitation, the plates were washed three times using PBST and blocked using 1% albumin in PBST for 1 h at room temperature. After another washing cycle, the plates were incubated overnight at 4° C with the IgG fraction purified from the *T*. *solium* cysts. A pool of sera from 15 cysticercotic pigs was also used in similar assays for comparison. Dilutions for both the IgG purified from the cysts and the pool of sera from the infected pigs are shown below. After washing, the plates were incubated with a HRP-coupled rabbit anti-pig IgG hyperimmune serum (diluted 1:1000) for 1h at room temperature. The reaction was developed using OPD (0.4 mg/mL) for about 3–5 min and stopped with 3N HCl. Absorbance at 492 nm was determined in a Multiskan FC (Thermo-Fisher Scientific).

In the case of western-blotting, 20 μg of the VF and the insoluble fraction were resolved through 12% SDS-PAGE and transferred onto a nitrocellulose membrane. The membranes were blocked overnight using 10% of skim milk in PBS and incubated with 10μg/mL of the IgG purified from *T*. *solium* cysts in 10% skim milk at room temperature. After three washings using PBS-Tween 0.1%, the membrane strips were incubated with the rabbit anti-pig IgG secondary antibody (diluted 1:85,000) for 2h at room temperature. The antigen-antibody reaction was developed using a West femto chemiluminiscence kit (Thermo) following the manufacturer’s instructions.

### Multi antigenic peptide testing

For each subset of *k* antigenic peptide measures and n subjects, predictive accuracy was measured by Leave One Out Cross Validation. Here, *n* independent training/testing procedures using a Support Vector Machine (SVM) were performed. Each training set consisted in all except one individual value, and testing set being the individual left out. Accuracy is computed as the fraction of times each individual test was correctly classified for that particular selection of *k* peptides. Source code for SVM implementation is found in S1 Data and is contained in scikit-learn package [SKLEARNREF] with default parameters [57].

### Tissue immune-localization studies

Cysts (CNS and SM) were fixed in Zamboni solution. Afterwards, all samples were dehydrated and embedded in paraffin. Heat-mediated antigen retrieval was performed on 5-μm sections, using a 0.1 M sodium citrate solution (pH 6.0) in a high-pressure sterilizer (120°C for 5 min) and endogenous peroxidase was consumed by incubation with 0.3% (v/v) H_2_O_2_ in PBS for 10 min at RT. Afterwards, the tissue section on slides were washed three times with PBS and maintained in a blocking solution (0.1% BSA in PBS, Sigma-Aldrich) for 10 min. After washing with PBS, the slides were incubated overnight with the primary antibody (the list of antibodies used could be found in S1 Table), diluted 1:50, in PBS-0.1% BSA, at 4°C. After washing several times with PBS, the tissue sections were incubated with the corresponding second antibody (HRP-conjugated) 1:1000 for 30 min at 37°C. Peroxidase activity was visualized by incubating the samples for 2 min with 3-diaminobenzidine tetrahydrochloride (DAB, MP Biomedicals). Reaction was stopped with water, and sections were counterstained with hematoxylin, dehydrated, cleared, and mounted with resine (Gold Bell). The single labeled sections were examined and photographed under light microscopy (Nikon Eclipse 80i) using a digital color video camera (Nikon Digital Sigth). The second antibody controls could be found in S3 Fig.

### Data availability

All relevant information are within the manuscript or supplementary material. The mass spectrometry proteomics data have been deposited to the ProteomeXchange Consortium via the PRIDE [58] partner repository with the dataset identifier PXD00527. The source code for the support vector machine is available in the S1 Data; protein and peptide identifications can be found in S2 and S3 Data; the subset of highly antigenic proteins can be found in S4 Data; the functional annotation of the tissue-enriched proteins in S5 Data and several cestode proteome comparison in S6 Data.

### Western-blotting of host proteins in the cysts protein extracts

Briefly, 50 μg of VF and insoluble fraction of cysts tissue were resolved by 12% SDS-PAGE, the gels were transferred onto nitrocellulose membranes. The membranes were blocked overnight using 10% of skim milk in PBS and incubated with the primary antibody diluted 1: 3000 in 10% skim milk at room temperature (S1 Table). After three washings using PBS-Tween 0.1%, the membrane strips were incubated with the corresponding secondary antibody diluted 1:50,000 in PBST. The antigen-antibody reaction was developed using a West femto chemiluminiscence kit (Thermo) following the manufacturer’s instructions.

### Proteome comparisons

We compared our proteomic dataset with several comprehensive proteomic studies performed in *Echinococcus granulosus* [26], *E*. *multilocularis* [27], *Mesocestoides corti* [28] and the theoretical secretome of *T*. *solium* [59]. In the case of *Echinococcus* and *Mesocestoides* proteomes, the sequences of the identified proteins in those studies were obtained from WormBase (www.parasite.wormbase.org/). After, those sequences were blasted against the *T*. *solium* database (http://www.genedb.org/blast/submitblast/GeneDB_Tsolium). Then, the top-ranked protein was considered the *T*. *solium* homologue of a certain *Echinococcus* or *Mesocestoides* protein (the complete list can be found in S6 Data).

### Ethics statement

The Animal protocol was revised and approved by the Ethical Committee for the Care and Use of Laboratory Animals at the Institute for Biomedical Research, Universidad Nacional Autónoma de México, under the license number ID 199 which follows the guidelines stated by the National Institutes of Health Guide for the Care and Use of Laboratory Animals.

## Results

### High-throughput proteomics of *T*. *solium* cysts

The proteome of the larval phase of *T*. *solium* was a complex mixture of parasite and host proteins. Using a TMT-multiplexed strategy, we were able to identify and quantify over 4,200 proteins across the nine cyst samples. Among these proteins, 3,368 were identified as parasite proteins, whereas 891 proteins were of host origin (Fig 1A). To our knowledge, this is the largest number of identified and quantified proteins in a multiplex assay for a cestode parasite to date. All the proteins were found across all the samples. More than 99.4% of proteins (including host or parasite) were identified in the nine samples. However, in the case of proteins TsM_000997700 and TsM_000195700, single peptides were only present in 8 samples and absent in only one. The protein changes observed between parasites obtained from different hosts and tissues were relatively discrete; the large majority of the identified and quantified cyst proteins remained at similar levels of expression, i.e., more than 3,200 proteins were found within a fold change of -1:1 (Fig 1B and 1C) for the SM and CNS cysts of the five pigs analyzed. Quantified host proteins were more variable between cysts from different tissues than between cysts from different pigs, thereby supporting the reproducibility of protein changes across the study (Fig 1B and 1C).

However, several parasite and host proteins were enriched for certain tissue localization of the cysts. For example, protocadherin alpha 6 (P = 0.0003), actin type 5 (p = 0.0002), a component of the γ-tubulin complex (P = 0.0001), a subunit of the splicing factor 3A (P<0.0001) and a protein associated with microtubules (P = 0.0001), were more abundant for cysts dissected from SM. On the other hand, proteins significantly associated to cysts dissected from the CNS of the pigs included a DNA topoisomerase 1 (P = 0.0001), alanine aminotransferase (P = 0.0002), an aldo-keto reductase (P = 0.0002), dnaJ protein (P = 0.0001), a protein containing a carbohydrate kinase domain (P<0.0001) and two hypothetical proteins (P<0.0001 and P = 0.0003) (Fig 1D). Two groups of host proteins were also found enriched for each tissue localization of the cysts (Fig 1E).

### Host proteins in the cysts

Several studies consistently detected intact host proteins in protein extracts of Taeniid parasites [30, 60–64]. In a recent report, we identified 17 host proteins in the vesicular fluid of *T*. *solium* cysticerci [63]. The results reported here, to our knowledge, are by far the largest set of host proteins reported within the tissues and fluids for any cestode parasite, suggesting a highly complex and close contact between the porcine and the cysts proteins.

**Fig 2:**
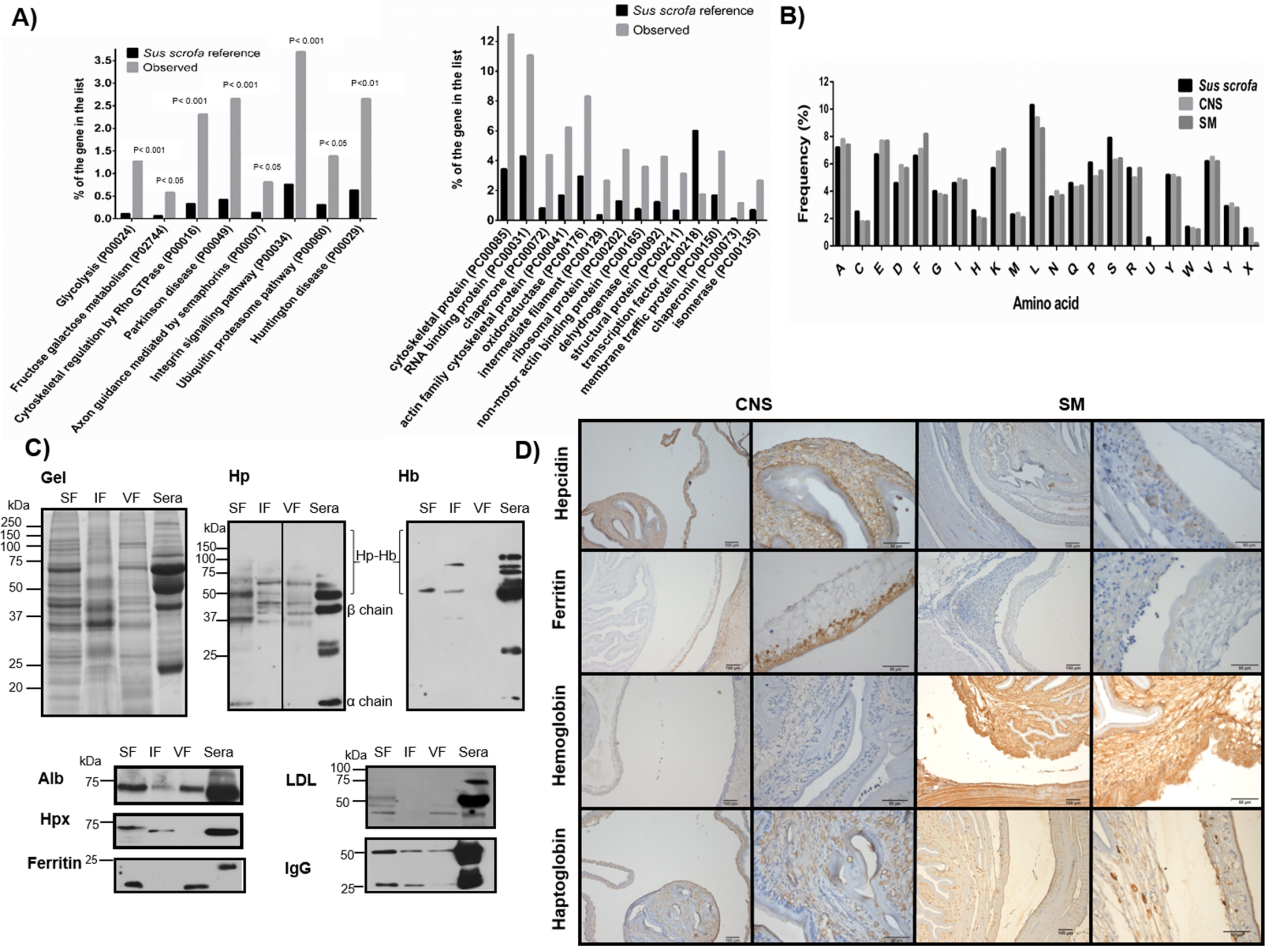
Annotation, composition and localization of host proteins in *T*. *solium* cysts. A) Significantly enriched host proteins in cysts extracts: metabolic pathways (categories with a P value<0.05) and molecular functions (categories with a P value <0.001). B) Amino acid composition of the host proteins associated with the central nervous system (CNS) and the skeletal muscle (SM) localization of the cysts (P value< 0.05), for comparison is shown the amino acid composition of the whole *Sus scrofa* proteome. C) Using specific antibodies several host proteins were identified in cyst’s protein extracts. D) Immuno-localization of host proteins related to iron metabolism in cysts obtained from the CNS and SM; scale bars in the first and third column of images correspond to 100 μM whereas in the second and fourth columns correspond to 50 μM.

It has been proposed that the host proteins are up-taken through a non-specific mechanism such as fluid phase endocytosis [60]. In our dataset, the host proteins were more variable than cysts proteins, this could be associated with a differential composition of the cysts microenviroment, CNS vs SM (S2 and S3 Data). Gene ontology analysis allowed determining that a diversity of host proteins were present; categories included metabolic enzymes involved in pathways like glycolysis or fructose/galactose metabolism, as well as signaling proteins including those participating in the integrin and ubiquitin-proteasome system. Functions of the uptaken host proteins included RNA binding, chaperones, oxidoreductases, ribosomal and isomerases (Fig 2A), those pathways were also enriched for the skeletal muscle proteome of the pigs [65], suggesting that the up-taken host proteins reflect the composition of the cysts micro-environment (S5 Data).

It has been proposed that the cysts use the up-taken host proteins, including immunoglobulins, as a source of amino acids [66, 67]. The amino acid composition of the host proteins resulting from cysts obtained from CNS and SM was very similar; the biggest differences were found for aspartic acid and serine, reaching only an increase of 1.2% and a decrease of 1.5%, respectively (compared with *S*. *scrofa* proteome). The similar amino acid composition of up-taken host proteins in CNS and SM cysts is consistent with the concept of an unspecific mechanism for the host protein uptake by the cysts (Fig 2B).

We also explored the question of the host proteins tissue localization. Tissue localization studies were carried out to determine the distribution of several host proteins within the cyst’s tissues. We selected host proteins related to iron metabolism in the host: haptoglobin (Hp), hemoglobin (Hb), hemopexin (Hpx), hepcidin and ferritin; other host proteins such as LDL, albumin and IgG, were used for comparison. Two experimental approaches were employed: cyst protein fractionation followed by western blotting and immuno-localization in tissue-sections. Through western blotting we found the majority of host proteins in the vesicular fluid, as well as in the soluble fraction of cyst’s tissue (Fig 2C). Abundant host proteins such as IgG and albumin were detected in the three protein fractions tested (soluble and insoluble fraction of cyst´s tissue and in the vesicular fluid). Hemopexin (Hpx) was found in the tissue’s extracts (soluble and insoluble fractions) and was not detected in the vesicular fluid; in the case of the insoluble fraction of cysts tissue, the immuno-reactive band was detected at the same molecular weight as the positive control (serum) and in the soluble fraction a band with a slightly increased molecular weight was detected. LDL was found present in the cyst’s tissue soluble protein extract and scarce in the vesicular fluid (Fig 2C).

In the case of the host proteins related to iron metabolism, the most abundant ones found in the cyst’s tissues were Hp and Hb; for Hp several bands were recognized (most of the bands were shared with the positive control), but others only appeared in the cysts extracts. In the case of Hb, immunoreactive bands were detected in the cysts tissue. After immune-histochemical analysis we found a conspicuous distribution of Hp and Hb in the cyst’s tissues, particularly in tissue surrounding the spiral canal of the invaginated scolex. Hepcidin and ferritin were also found in the cyst’s subtegumentary tissue, but in the case of ferritin in cysts extracts, the immuno-reactive bands had lower molecular weight than the band in serum (Fig 2C and 2D). All tested host proteins (with the exception of ferritin) were detected in their expected molecular weight in gels, suggesting that at least a fraction of the protein is intact.

**Fig 3:**
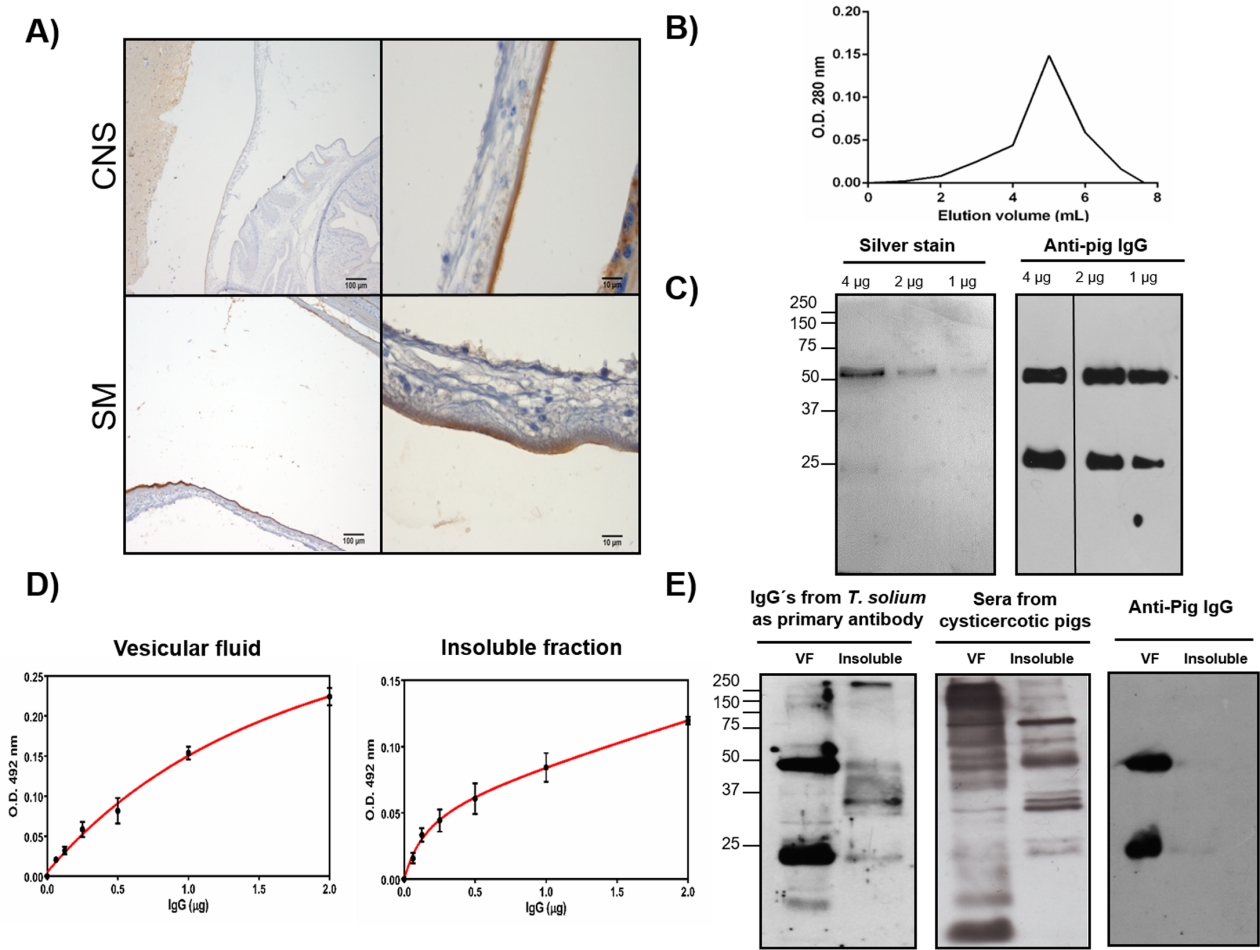
Immuno-localization and functional evaluation of host immunoglobulins purified from protein extracts of *Taenia solium* cysts. A) Immuno-localization of host IgG in cysts obtained from the central nervous system and skeletal muscle of pigs. B) Immunoglobulins present in total protein extracts of cysts were purified using a Sepharose 4B column coupled with Protein G. C). The purity of the bound IgG was evaluated by SDS-PAGE and western blotting using an anti-pig IgG coupled to HRP. D) The antibody activity of purified IgG was evaluated by ELISA. Samples of *T*. *solium* vesicular fluid and insoluble fraction were used to coat 96 well microtiter plates. Afterwards, the purified IgG was incubated overnight at 4°C under slow agitation. Then, the antigen-antibody reaction was developed using a colorimetric method. E) Recognition of cysts proteins by the purified IgG through western blotting. Samples of *T*. *solium* vesicular fluid and insoluble fraction were resolved through SDS-PAGE and then transferred onto a nitrocellulose membrane. The purified IgG from cysts protein extracts was used as primary antibody (for comparison sera from cysticercotic pigs was also included) and an anti-pig IgG coupled to HRP was used as secondary antibody. Lower right panel shows the reaction with the secondary antibody alone as control.

This idea was explored further using as a model the uptaken host IgG. IgG was located on the outer surface of cysts in both CNS and SM cysts, with a more intense signal in SM cysts (Fig 3A). This localization was consistent with a previous report [68]. To explore the antibody activity of the uptaken host IgG; we purified host IgG from total saline extracts of SM cysts through affinity chromatography using protein G (Fig 3B). The purity of the isolated pig IgG was evaluated by SDS-PAGE and western blot (Fig 3C). Heavy and light IgG chains were detected at the expected molecular weights (50 and 25 kDa), suggesting that pig IgG was uptaken intactly. Two assays were performed to test the recognition of *T*. *solium* antigens by the purified pig IgG: ELISA and western blot using vesicular fluid (VF) or the insoluble fraction of the cysts as parasite antigens and the purified IgG as primary antibody (Fig 3D). The purified pig IgG reacted both, with the VF and the insoluble fraction of cysts antigens in a saturable and specific way. For western blotting, both antigenic fractions, VF and the insoluble fraction reacted with the purified IgG from the cysts (as shown in Fig 3E). As expected, several bands were recognized and were shared with the band obtained using sera from cysticercotic pigs (Fig 3D), indicating that uptaken host immunoglobulins retained their antigen-binding activity.

### Proteomic changes of *T*. *solium* cysts obtained from SM and CNS

**Fig 4:**
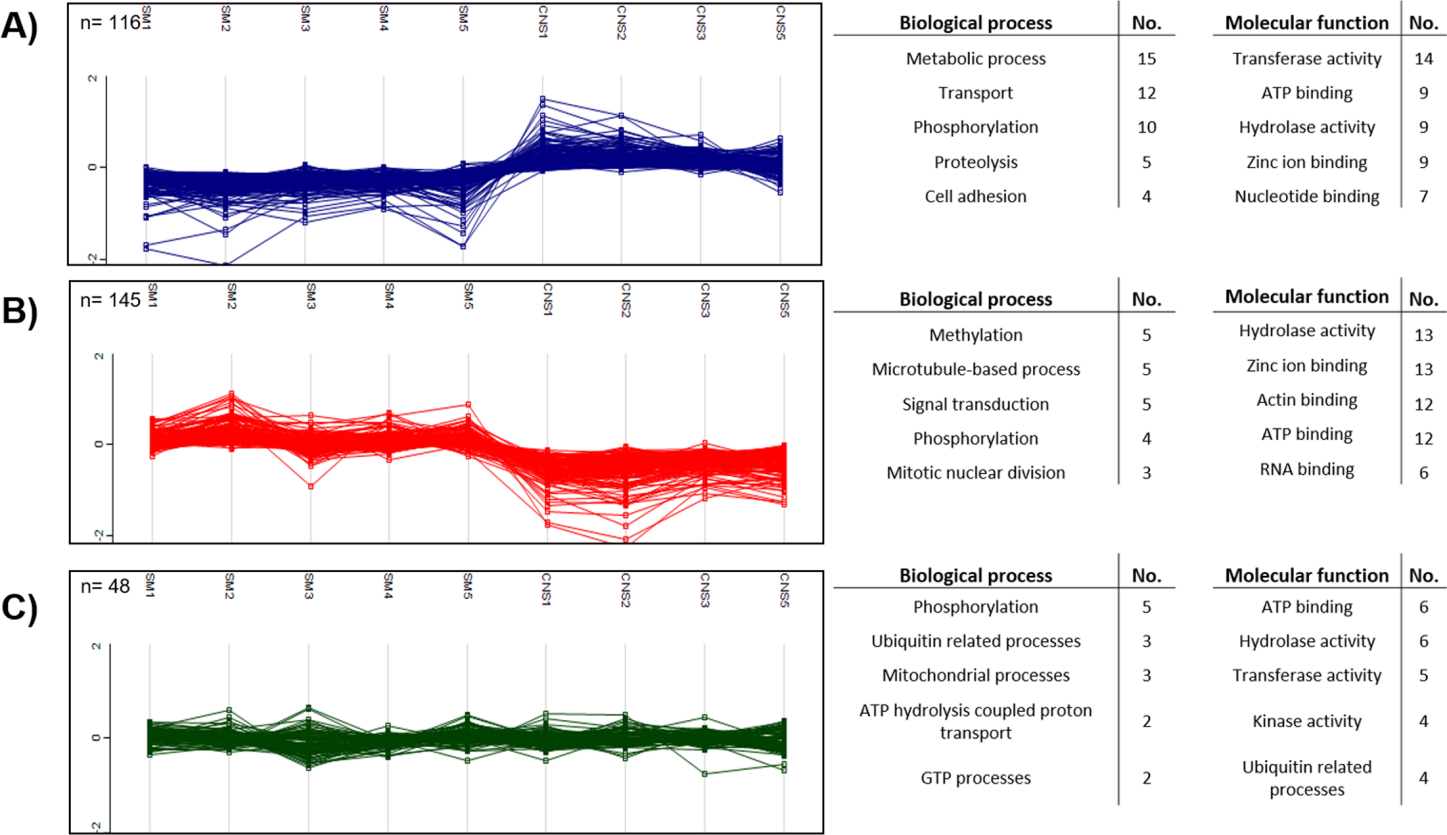
*k*-means clustering and associated biological/molecular functions for proteins of skeletal muscle (SM) and central nervous system (CNS) cysts. Cluster associated with the CNS (A), SM localization (B) and 48 constitutive proteins with the lowest coefficient of variation (C). The proteins included have a P-value <0.01 (n = 261). Only the most frequently observed categories and molecular functions (obtained using Argot2 algorithm) are show on the right panel. The y axis shows the Log_2_ (relative abundance).

To explore the molecular functions and biological processes of the *T*. *solium* proteins identified for each tissue localization, we performed a *k*-means clustering analysis using a subset of cysts proteins with a significant fold change (P-value<0.01, n = 261). Two major clusters were identified, one for each tissue localization (Fig 4). For CNS cysts (Fig 4A), 116 proteins were abundant for parasites of this tissue localization (compared with SM parasites). These proteins were associated with metabolic processes, transport and phosphorylation. In the case of SM cysts (Fig 4B), methylation, signal transduction and microtubule-based processes were the most frequently observed categories. For comparison, 48 proteins with the lowest coefficient of variation that remained in similar levels between cysts from different tissues were selected, including proteins associated with ubiquitination, phosphorylation and mitochondrial processes (Fig 4C, S5 Data). The relevance of those pathways in explaining both preferential localizations of the cysts, in terms of adaptation and survival within the host tissues, deserves future study.

**Fig 5:**
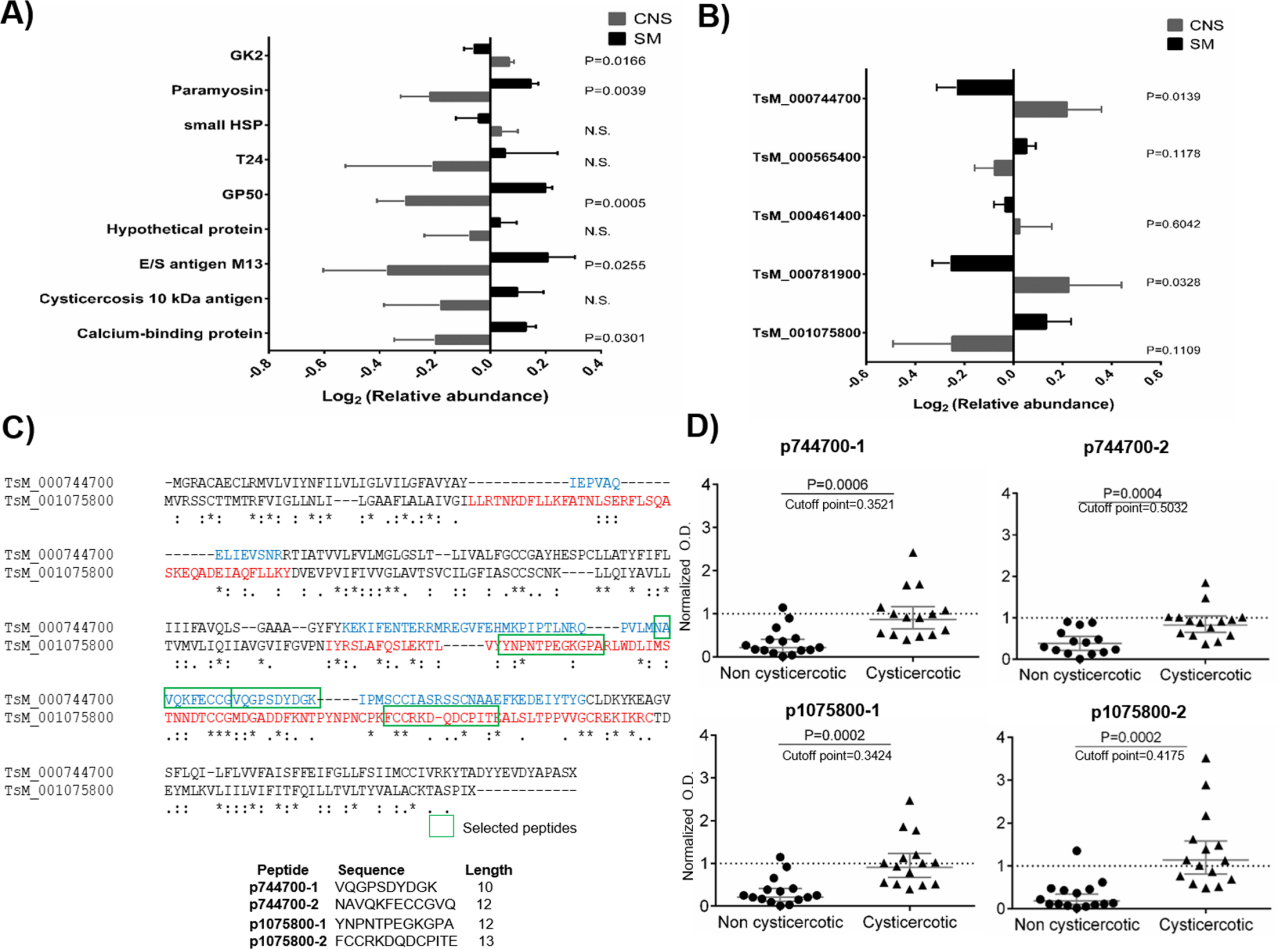
Protein abundance of several well-studied antigens in *T*. *solium* cysts antigens. A) Relative abundance of cyst’s antigens obtained from the skeletal muscle (SM) and central nervous system (CNS); B) Relative abundance of tetraspanin proteins quantified in our proteomic analysis for SM and CNS cysts; C) Alignment of SM- and CNS-abundant tetraspanins. The extracellular loops are bold in red and blue, selected peptides are marked by a square. Listed at the bottom are the selected peptide sequences and length. D) Recognition of the synthetic peptides by non cysticercotic and cysticercotic pig sera. The normalized optical density (O.D.) is the result of dividing each individual O.D. by the cut-off value (mean value of non cysticercotic pigs plus two standard deviations). P-values are shown at the top of each figure.

As mentioned above, several host and parasite proteins were associated with the tissue localization of the cysts. To investigate more thoroughly that possibility, several previously described antigens (reviewed in [69]) were queried for in our database. As shown in Fig 3, several relevant antigens in cysticercosis (*i*.*e*., paramyosin, GP50 and a calcium binding protein), were more abundant in SM cysts than in CNS cysts (Fig 5A). Moreover, we mined our database to search for other antigens enriched for each tissue localization. The case of the tetraspanin family appeared to be especially interesting as tetraspanins are relevant antigens in schistosomiasis [70] and hydatidosis [71]. Our results showed the expression of five members of the tetraspanin family; of these, two proteins were enriched in the CNS localization and one in the SM localization of the cysts, while the other two have no detectable changes. These proteins: TsM_000744700 and TsM_001075800 were subsequently analyzed (Fig 5B). Initially, the large extracellular loop of these proteins was deduced using several algorithms (see Materials and Methods). In those tetraspanins, the protective domain (the protein region associated with protection in vaccination trials) is located within the large extracellular loop [70, 71]. Therefore, this portion of the protein was analyzed for antigenicity using algorithms for B cell epitope-prediction. Two peptides were chosen from each protein and synthesized through a commercial service (TsM_000744700: VQGPSDYDGK, NAVQKFECCGVQ; TsM_001075800: YNPNTPEGKGPA, FCCRKDQDCPITE) (Fig 5C). To explore if these four peptides (p744700-1 and 2; p1075800-1 and 2) were recognized by antibodies in the sera of cysticercotic pigs, two groups of 15 sera (cysticercotic and non cysticercotic) from pigs bred in rural endemic areas were used. As shown in Fig 5D, the four peptides were recognized by several infected animals, with the peptide FCCRKDQDCPITE (p1075800-2) having the strongest antibody recognition.

**Fig 6:**
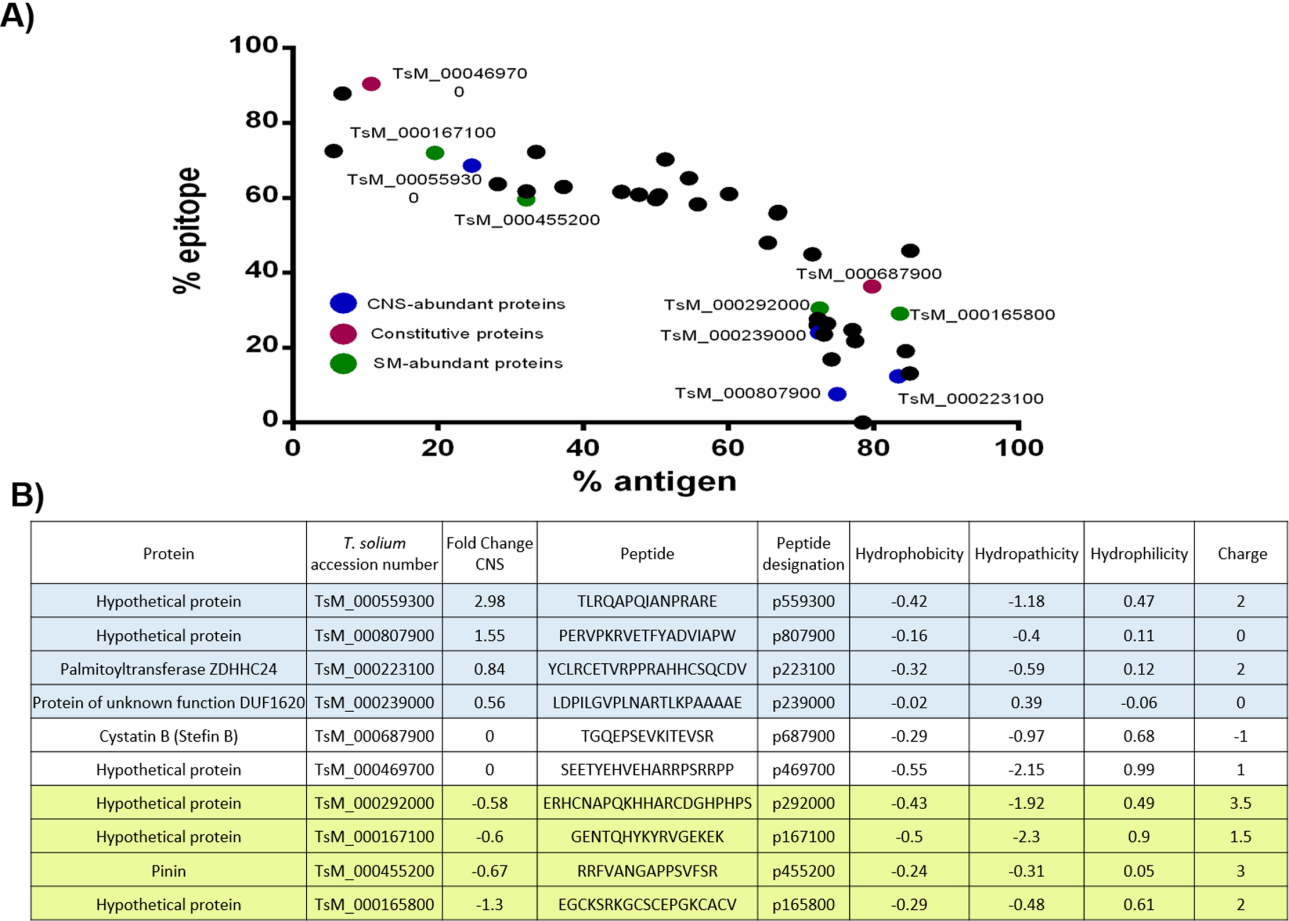
Selected peptides from skeletal muscle (SM) and central nervous system (CNS) abundant cysts proteins. A) Proteins predicted with high antigenic and/or epitope content. B) Ten proteins were selected: four SM (green) and four CNS (blue) abundant proteins. Two constitutive proteins were also chosen. One peptide was then selected for each protein.

The differential abundance of several antigenic proteins (GP50s, paramyosin, E/S protein M13, calcium-binding protein, tetraspanins, etc.) between SM and CNS cysts, provides evidence about the presence of antigens that were enriched for SM or CNS cysts. Our next goal was the identification of highly antigenic tissue-enriched proteins; here we defined a tissue-enriched protein as one with differential abundance between CNS and SM cysts (p value<0.01). The high antigenicity was defined using B cell epitope and antigenicity predictors, see Materials and Methods. First, we selected 261 proteins with a significant fold change (P value<0.01) and 48 proteins with the lowest coefficient of variation, for comparison. Those proteins were analyzed through several antigenicity algorithms (see Materials and Methods and S2 Fig). Several proteins were predicted as strongly antigenic by one algorithm (antigen/epitope content ≥ 70%). Another group of proteins was also predicted as highly antigenic by both algorithms, although with a lower antigen/epitope content (50%) (Fig 6A).

Using this approach, 40 highly antigenic proteins were identified (the complete list of proteins can be found in S4 Data). To experimentally test the reliability of our antigenic prediction, 10 proteins were selected (Fig 6A and 6B). Of these proteins, four were enriched for CNS cysts, four were enriched for SM cysts and two proteins with a low coefficient of variation between CNS and SM cysts, none of these proteins had been studied before in *T*. *solium*. Then, a single epitope was selected for each protein (selected epitopes had to be predicted by both algorithms). After the synthesis of the antigenic peptides (Fig 6B), they were evaluated for antibody recognition by ELISA, using the same two groups of sera mentioned above. For a peptide to be considered as a valid antigen, the difference between the recognition of the cysticercotic and the non cysticercotic pig sera had to be statistically significant.

**Fig 7:**
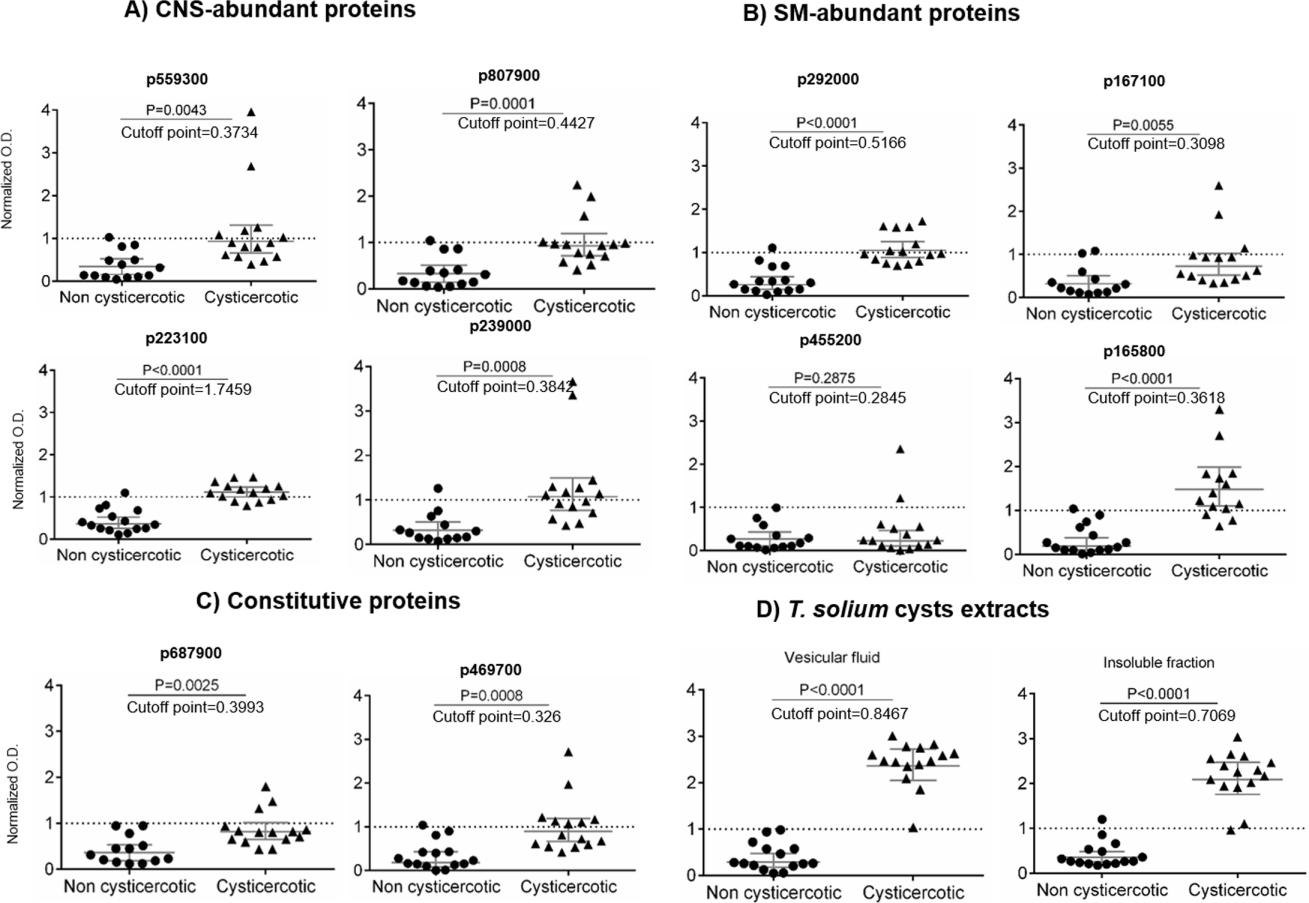
*Taenia solium* peptide recognition by cysticercotic and non cysticercotic pig sera through ELISA. A) Peptides from central nervous system, B) skeletal muscle abundant proteins, C) constitutive proteins and D) cysts protein extracts. Microtiter plates were coated with the peptides or extracts and incubated with sera from non cysticercotic (n = 15) and cysticercotic (n = 15) pigs, all bred in rural endemic areas. The normalized optical density (O.D.) is the result of dividing each individual O.D. by the cut-off value (mean value of non cysticercotic pigs plus two standard deviations). P-values are shown at the top of each figure.

Nine of 10 peptides (with the exception of one based on pinin) were significantly recognized by the sera from cysticercotic, in comparison with the sera from non cysticercotic pigs (Fig 7). These data, suggest that the proteins from which the peptides were originated are immunologically recognized in porcine cysticercosis; then we have identified several tissue-enriched antigens; interestingly, a peptide that was enriched for SM (p165800) and other for CNS (p223100) cysts, produced the highest difference between the cysticercotic and non cysticercotic pig sera (Fig 7A and 7B). The recognition by the IgG present in the sera of cysticercotic pigs, suggested that there is a subset of tissue-enriched antigens for cysts located in different host tissues. However, whether the rest of the predicted antigenic proteins are valid antigens in cysticercosis requires further screening.

### Diagnostic potential of tissue-enriched antigens

**Fig 8:**
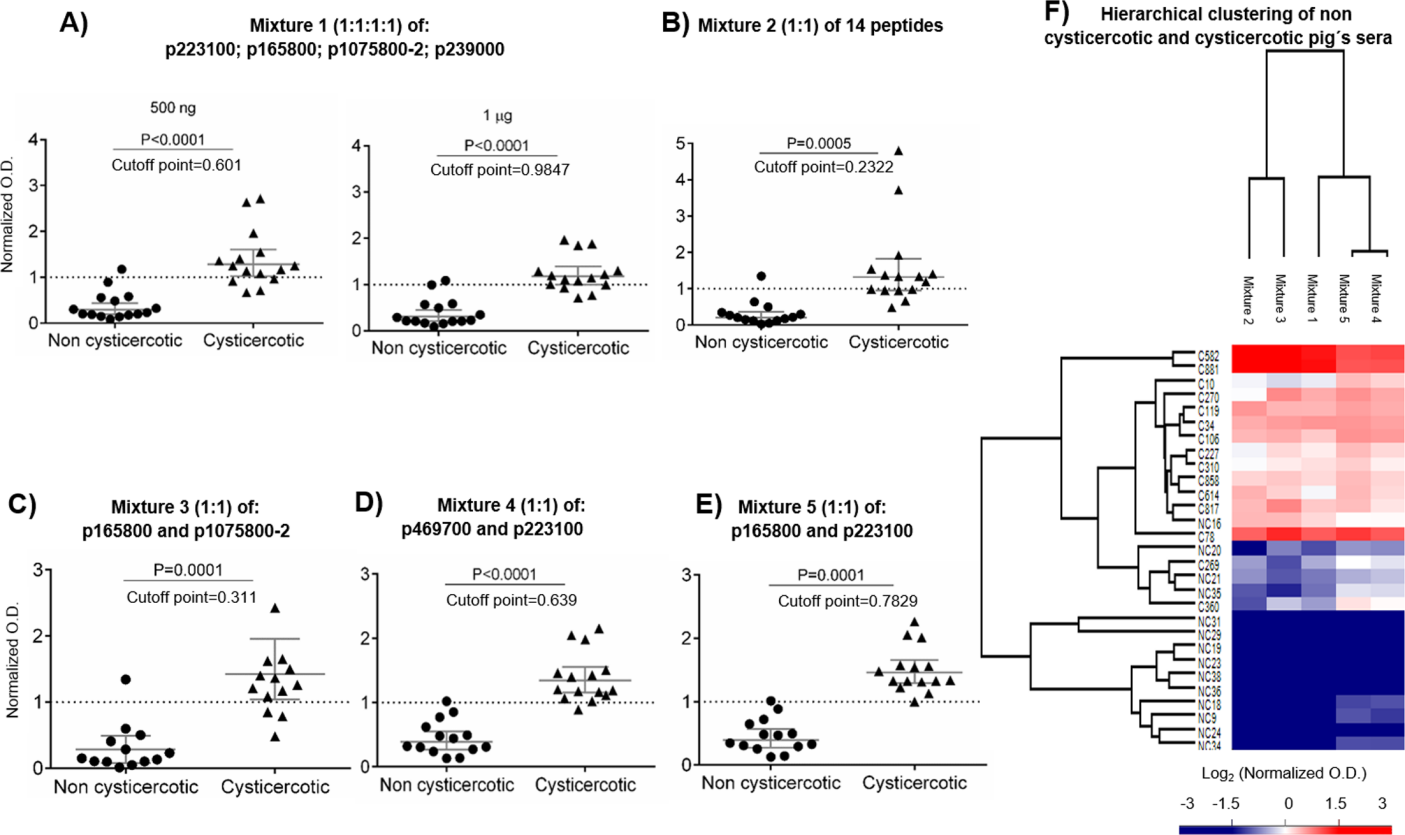
Peptide mixtures as potential diagnostic agents for *Taenia solium* cysticercosis. Several peptide mixtures were used: A) The best four individual peptides (using 500 ng or 1 μg to coat each microtiter plate well). The rest of the peptides combinations were used at 500 ng. B) Mixture of all 14 peptides synthesized in this study, C) Mixture of the peptides derived of skeletal muscle (SM) abundant proteins, D) Mixture of one peptide of a central nervous system (CNS) abundant protein and one of a constitutive protein and E) Mixture of peptides from SM and CNS cysts. The normalized optical density was calculated by dividing each individual O.D. by the cut-off value (mean value of non cysticercotic pigs plus two standard deviations). P-values are shown at the top of each figure. F) Heat map showing the individual response to antigenic peptide mixtures. The normalized optical density was transformed using Log_2_. White represents values near to the cut-off point, red represents values over the cut-off point (positive samples) and blue represents values below the cut-off point (negative samples).

The tissue-enriched antigens could be exploited for the improvement of current diagnostic tools for cysticercosis. A diagnostic test for cysticercosis would ideally include antigenic determinants for each possible tissue localization of the cysts, as well as antigens that are not affected by the tissue-localization of the cysts. To explore the diagnostic potential of those tissue-enriched antigens, several combinations of peptides were used in mixtures. The initial mixture was made using the peptides that were previously found to produce the highest optical densities, when tested separately with the same cysticercotic pig sera: p223100, p165800, p1075800-2 and p239000 (1: 1: 1:1); two concentrations were employed (Fig 8). As shown in Fig 8A, the lowest concentration produced better results. However, not all sera from the cysticercotic animals produced a significantly positive reaction when compared with the non cysticercotic pig’s sera. Other combinations of synthetic peptides were also tested, including a mixture of the 14 peptides that produced the worst performance (Fig 8B). We also tested combinations of peptides from SM-abundant proteins (Fig 8C), or/and SM constitutive proteins (Fig 8D). Interestingly, when the mixture included the best peptide for each tissue localization, p223100 for CNS and p165800 for SM, 14 out of 15 cysticercotic and non cysticercotic pig sera were clearly differentiated (Fig 8E). However, we were not able to improve the performance obtained using the two crude protein extracts from the cysts, indicating that the 'ideal' antigenic subset will require further investigation.

In this study, each pig showed a differential response for each peptide and peptide mixture (Fig 8F); the idiosyncrasy of individual humoral host immune responses against *T*. *solium* cyst antigens is well known [3], as it is in other infectious diseases [72–74]. In addition, the considerable genetic/antigenic variation between cysts obtained from different endemic areas it is frequently reported [75–77].

The immuno-diagnosis of an infectious disease is often performed using a single antigen, i.e., using a single protein/peptide or antibody to discriminate between healthy and infected hosts. In the case of infections caused by *E*. *granulosus*, the use of AgB and 8 kDa proteins have been tested as diagnostic agents. However, new methodological approaches are needed for parasite infections such as schistosomiasis, echinococcosis and cysticercosis, to discriminate between infected hosts with low-parasite loads [1, 2; 78, 79].

A novel approach involves the machine-learning models that have proven useful in the diagnosis and prediction of several diseases. Several algorithms have been developed for the diagnosis of breast [80], colorectal [81] and non-small cell lung cancer [82]. Distinct antigenic response patterns (ARP) may constitute better representations of the pathogen’s fingerprints than single-peptide responses. Thus, a multi-antigenic peptide testing (MAPT) can identify such ARP for each infection.

To explore the viability of a MAPT using our synthetic peptides, we constructed an antigen response space (ARS), where each individual is represented by a single point. The position of each individual point (one pig’s serum) depends of its antigenic response to several peptides. For *k* antigenic peptides considered, *k* OD measures determine the individual position in ARS. In this sense, an ARP can be defined as a particular region in ARS where all infected hosts are present. Machine-learning algorithms can be directly used to identify boundaries of such regions. It should be noticed that a given ARS could represent a corresponding antigenic peptide subset.

**Fig 9:**
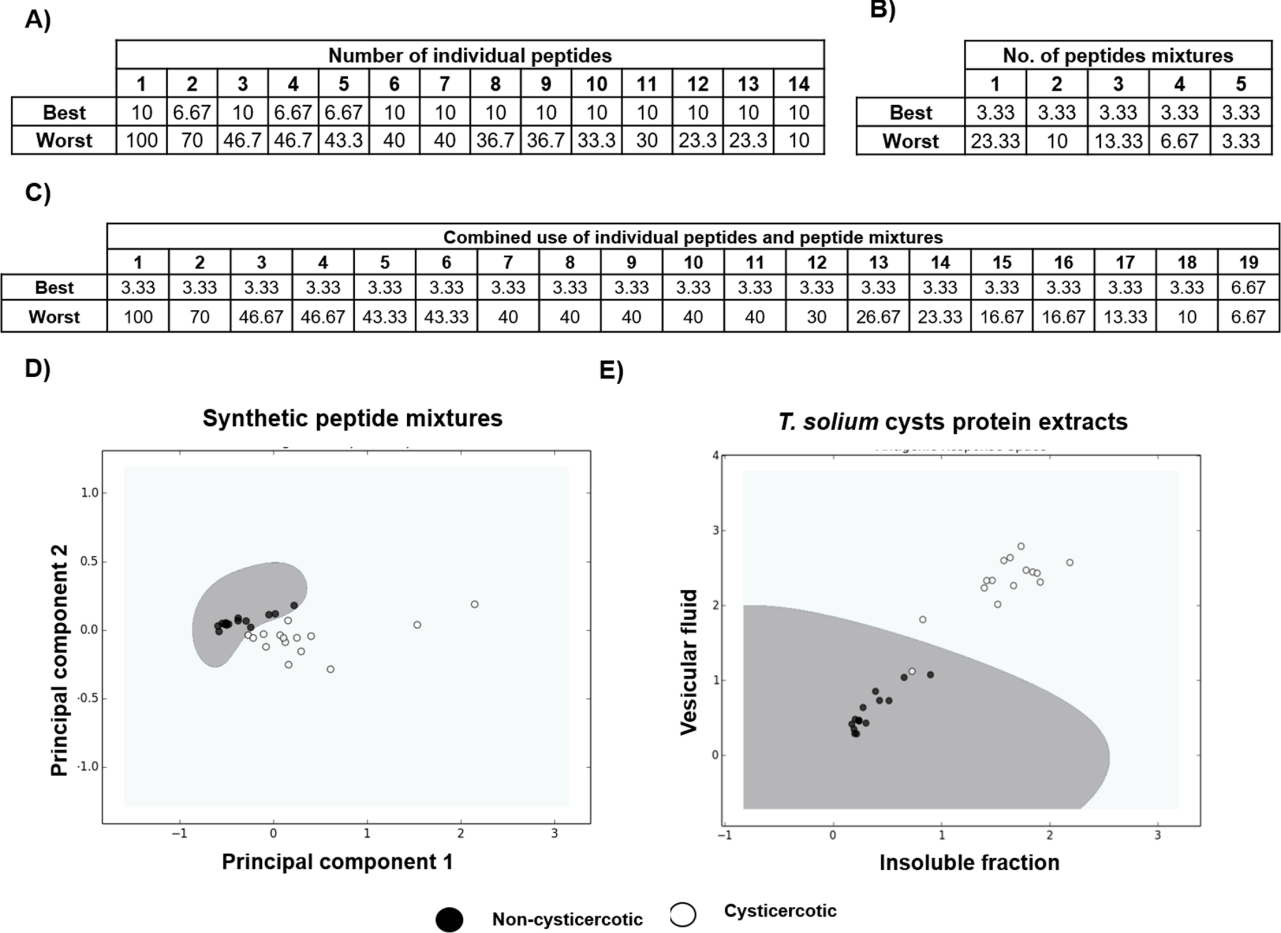
Antigenic response space. The raw optical densities were analyzed using machine-learning methodologies. We determined the performance of (A) individual peptides measurements, (B) peptide mixtures measurements and (C) the combination of both strategies. Best/worst refers to the percentage of error (taking into account our n = 30; an error of 10 implies that three pig sera were not discriminated). Visual representation of the antigenic response space for multiple antigen testing (D) and *T*. *solium* cysts protein extracts (E).

To explore the potential of the synthetic peptides to define an ARS for pig’s cysticercosis, all possible combinations of peptides were evaluated. Analysis was performed taking 14 single peptide measures, 5 peptide mixtures measures, and a combination of both. Thus, 16,383, 31 and 524,287 possible ARS representations were evaluated in each case. Evaluations were performed with leave-one-out cross validation and a support vector machines as a classifier (see Materials and Methods). Number of errors achieved (expressed as percentage) by the best and worst combination for all possible number of peptides were used simultaneously for ARS constructions. As depicted in Fig 9A–9C, the use of peptide combinations usually produced better results than individual peptides. For comparison, we produced an image of ARS using peptide combinations and cysts protein extracts (Fig 9D and 9E). Using this approach we were able to discriminate infected versus non-infected pigs. This discrimination resulted in a similar performance to the one obtained using complex crude cysts extracts (Fig 9D and 9E).

Based on our current preliminary results, developing an accurate immunodiagnostic test for cysticercosis, requires a number of specific antigens from cysts of different tissue localizations within the host, as well of antigens from cysts obtained from different geographical areas. In addition, the use of novel-analytical tools such as machine-learning models, can efficiently discriminate between healthy and infected hosts.

In contrast, we tested this approach using sera from non-cysticercotic (n = 8) and neurocysticercotic patients (n = 12). As expected, our ARS approach produces better results than using a single peptide or peptide mixture. However, sensitivity was about 75% (S4 Fig) indicating that selection of adequate peptides/proteins for diagnosis in humans will require separate studies.

## Discussion

Several studies have recently focused on deciphering the proteome in *Taeniid* parasites. The proteomes of the whole larva, the protoscolex, the pre-adult stage and some immunogenic proteins have being characterized for *Echinococcus spp*. [83–87]. The proteome of *T*. *solium* has been less explored, although the composition of the excretion/secretion products [88], the proteins of activated oncospheres [89] and a small group of immunogenic proteins have been reported [62]. Moreover, an algorithm for the identification of unique mass spectra for *Taeniid* parasites has been developed [90]. In addition to the fact that these are still initial efforts, a relevant aspect that remains uncharacterized refers to the proteomic changes associated with the tissue localization of the cysts in the host. Knowing these changes might be essential to understand the tissue preference of the cysts. Although *T*. *solium* cysts can establish in a variety of tissues they appear to show preference towards the skeletal muscles and several locations in the central nervous system, including the brain.

In this report, we describe the proteome of *T*. *solium* cysts obtained from CNS and SM of infected pigs. We used state-of-the-art quantitative isobaric proteomics to identify and quantify more than 4,200 proteins in a single assay. This is the largest number of identified and quantified proteins so far described for a cestode parasite. A challenging finding is the high amount of host proteins in all crude extracts of *T*. *solium* cysts [60–63]. The presence of intact host proteins in the extracts from cestode parasites has been known for six decades [60–64, 91]. Some recent reports on *Echinococcus spp*. proteomics have also identified a variety of host proteins in the protein extracts of this cestode, for example, 43 proteins were identified in the cysts fluid [27] and up to 293 proteins were identified as excretion/secretion (E/S) products [59]. In our study, we were able to quantify 891 proteins of host origin, the highest number of host proteins identified for a cestode parasite, which brings back the interest on the role of host proteins in the cyst’s physiology. However, if *Taenia spp*. contains more host proteins than *Echinococcus spp*. remains to be elucidated; both studies were performed using different proteomic (as well as sampling) strategies. Here, we benefited from high throughput and state-of-the-art multiplexed proteomics that enabled us to identify a significant number of cyst and host proteins.

Herein, one out of each five proteins identified resulted of host origin. These identified host proteins are involved in a number of metabolic, physiologic, signaling and regulatory processes for the pig. It is worth remembering that the *T*. *solium* genome revealed a greatly simplified organism, lacking a number of metabolic processes (biosynthesis of amino acids, fatty acids, etc.) as a result of its evolutionary adaptation to parasitism [14]. It is conceivable that the host proteins present in the cysts (associated with metabolic and signaling functions) could play a role for the parasite, beyond being a mere source of amino acids. In the case of *E*. *granulosus*, the identified host proteins were also associated with metabolic processes, response to stimuli and regulation of biological processes [26]. It is conceivable that some host proteins retain their function and could play a role on metacestode physiology.

In order to explore this idea, we carried out functional assays and tissue localization studies for a group of host proteins. For example, highly abundant host proteins like albumin and IgG were found in all protein fractions obtained from the cysts, indicating that their presence is ubiquitous in parasite’s tissues and fluid. Another example were LDL and hemopexin, which were found in the cyst’s tissue but were scarce in the vesicular fluid. Cestode parasites have a reduced capacity for lipid biosynthesis [14]. In the case of *Schistosoma mansoni* (trematode), several proteins have been identified as LDL-binding proteins [92, 93] and LDL was found associated with parasite’s tegument [94]. In our study, we found the LDL protein associated with cysts tissue. However, if *Taenia spp*. parasites have a subset of specialized proteins to bind and uptake LDL from the host remains to be seen.

Hpx was abundant in the cysts tissue extracts, while scarce in the VF; interestingly, in the soluble fraction of cysts tissue, the Hpx band showed a slight increase in the apparent molecular weight, while in the insoluble fraction the band was detected at the same molecular weight as in the control serum. In the case of Hp and Hb, several bands were detected (especially for Hp); this finding can be explained by the presence of Hp in different forms: the free form, as well as in complexes with Hb. Furthermore, some bands could be the result of Hp/HpHb complex degradation by cysts proteases. We have recently described that intact and functional Hp are present in the cyst’s tissue and could be associated with the iron uptake by the cysts [64]. Here we widen the scope of our investigation on the possible involvement of host proteins in the cyst’s iron metabolism. We carried out tissue localization studies for several host proteins associated with the iron metabolism (hepcidin, ferritin, hemoglobin and haptoglobin). The four proteins were detected in the cyst’s tissue, being Hb and Hp the most abundant and widely distributed within the cysts, suggesting that a relevant portion of iron uptake by the larvae might be supported by these host proteins.

Hepcidin is a master regulator of iron metabolism produced by the liver and its active form is a peptide of 25 amino acids. Hepcidin binds to ferroportin and induces its lysosomal degradation, thus decreasing the iron export by the target cell [95]. However, hepcidin signaling appears to be restricted to mammals [96]; at least, no reports about a cestode homologue ferroportin are available; future studies are need to explore the role (if any) of hepcidin in cestodes biology. On the other hand, ferritin is considered the major iron storage protein [97]. Using specific antibodies, we found ferritin present in the subtegumentary tissue of cyst bladder wall, suggesting that it could also play a role for the parasite, the bands that were detected in protein extracts of cysts had a decrease in the molecular weight (compared with the control). However, after searching the *T*. *solium* genome database for ferritin, we found that the cyst has two homologues with a very similar predicted molecular weight, therefore, host and parasite ferritins appears to be undistinguishable in molecular size (≈20 kDa). Therefore, it is possible that the immuno-localized ferritin is a combination of host and cysts ferritin stocks. Whatever the source of ferritin is, its localization in close contact with the host tissue suggests that cysts accumulates specialized molecules for iron storage.

Albumin appears to be involved in the maintenance of parasite’s osmotic pressure in the vesicular fluid, fulfilling a similar function to the role it plays for the host [61]. In addition, immunoglobulins have been proposed as a source of amino acids for the cysts [66, 67].

We determined the antigen-binding activity of host IgG purified from total protein cyst’s extracts through ELISA and western blotting, testing their ability to react with cysts antigens using two protein crude extracts. Purified IgG from the cysts showed a clear antibody activity through the recognition of several antigenic bands (those bands were also shared when sera from cysticercotic pigs were used), suggesting that at least a part of the uptaken host IgG were specific antibodies directed against cysts proteins.

Several other host proteins could also retain their function. Many other uptaken host proteins could play a physiological role, for the parasite. It could also be that potentially deleterious host proteins are simply removed from the host-parasite interface. Ascertain if the host proteins play functions in the physiology of the cysts is an open area of research that could disclose a number of unexpected results.

With respect to the *T*. *solium* cysts proteins identified and quantified in our proteomic assays, the changes found between CNS and SM cysts were discrete; more than the 90% of the identified and quantified proteins (>3,100) were grouped within a fold change of -1 and 1. A tissue-enriched protein pattern (including cysts and host proteins) was associated to each cysts tissue localization. These protein patterns could represent a homeostatic adaptation to the biochemical conditions in different tissue environments (SM vs CNS). Our dataset was compared with others previous proteomic reports for helminths [26–28; 59]. From our dataset, 167 proteins were considered excretion/secretion proteins (compared with the *T*. *solium* theoretical secretome [59]) (S6 Fig, and S6 Data). In addition, almost 30% of the *T*. *solium* gene products were considered ‘hypothetical’, meaning that those genes could not be functionally annotated. Among those hypothetical proteins, the expression of 357 proteins was validated here (S6 Fig and S6 Data). After comparison with the proteomes of *E*. *granulosus*, *E*. *multilocularis* and *M*. *corti*, 14 proteins were common to the four cestodes (S6 Data); those proteins included: a 14-3-3 family member, a fatty acid binding protein, a protein with EGF domain, a lactate dehydrogenase, etc. Indicating that the proteome of cestode parasites are usually highly complex mixtures of parasite and host proteins. The relative consistence of high amounts of host proteins and the relevance of those common proteins identified between the four cestodes need future investigation.

After this initial characterization, several antigens were found enriched in the SM localization of the cysts (paramyosin, a calcium-binding protein, E/S antigens, etc.).

Similarly, other proteins belonging to different families (tetraspanins and GP50s antigens) were also differentially found between CNS and SM cysts. Tetraspanins are integral membrane proteins directly exposed to the host [70, 71] and have been considered vaccine candidates in several helminth infections [98–100]. However, since tetraspanins are highly polymorphic, vaccination trials have produced controversial results [101]. For *T*. *solium*, the tetraspanin family has been poorly characterized, only one member (T24) with a good performance as a diagnostic antigen [102], has been described. In this report, five members of the tetraspanin family were quantified; two were associated with the CNS localization of the cysts and one with the SM localization. To investigate if those proteins are antigens during cysticercosis, two peptides were chosen from the amino acid sequence of either TsM_000744700 or TsM_001075800. The four peptides were recognized by antibodies occurring in the sera of naturally infected pigs (four pigs showed a significant recognition of both proteins). The strongest recognition was associated with p1075800-2, derived from the SM-enriched tetraspanin. Regarding *T*. *solium* T24 tetraspanin, glycosylation strongly influenced its antibody recognition [102]. It remains to be seen whether the antibody-recognition of the tetraspanins studied here can be increased using recombinant and glycosylated forms.

The diagnostic antigen GP50, is a GPI-anchored glycoprotein with affinity to *Lens culinaris* lectin, this protein is a promising candidate agent for the immunodiagnosis of NC. Nevertheless, it showed a poor performance when sera from patients having a single viable cyst in the CNS were tested [103, 104]. Two GP50 proteins were found among our proteomic data (S5 Fig). The “canonical” GP50 was associated with the SM localization of the cysts, while a “truncated” form was slightly increased in the CNS cysts. The truncated CNS-abundant GP50 lacked a predicted GPI-anchoring site, in addition to less disulfide bonds and predicted N-linked glycosylation sites. A diagnostic test based on the combination of these two GP50s deserves further study.

We hypothesized that several tissue-enriched antigenic proteins can be used as markers for the tissue localization of cysts. This idea was explored through a combination of theoretical and experimental approaches. Initially, 261 proteins with significant fold changes were selected for epitope prediction using two algorithms; 40 proteins with high antigenic or high epitope content were found enriched either in SM or CNS cysts. Ten proteins were then selected for experimental testing through the synthesis of a single antigenic peptide chosen for each protein. All peptides were recognized by antibodies in the sera of infected animals, but with great variability. When tested with sera from cysticercotic and non-cysticercotic pigs, 9 out of 19 peptides were significantly recognized (p value < 0.01) by the sera from infected animals. The only exception was the peptide derived from a pinin: p455200. These results support the existence of tissue-enriched antigens. The immunodiagnostic potential of those peptides was also tested: peptides were used alone or in mixtures for recognition by the same group of cysticercotic and non-cysticercotic pig’s sera. The best results were obtained with several peptide combinations; in fact, the best combination included peptides derived from SM and CNS-enriched cysts proteins. This can only be considered as an initial study about the potential utility of tissue-enriched antigens; future studies are being conducted using the full recombinant proteins to increase the sensitivity of the immunodiagnostic tests.

A number of antigens have been tested as diagnostic agents. We hypothesized that testing multiple antigens, could produce a better strategy to distinguish between healthy and cysticercotic pigs. A machine-learning strategy was employed and 540,701 combinations were analyzed. Peptide mixtures produced better results than individual peptides. Using five mixtures of peptides allowed to discriminate between cysticercotic and non cysticercotic sera. Our results suggested that, at least five different antigenic determinants are required in order to develop an efficient diagnostic test, able to differentiate between the two groups of sera. Unfortunately, as those synthetic peptides were tested with sera from naturally infected pigs, we do not have relevant information about those infections, such as primary vs secondary infection, co-infections, and nutritional status.

The tissue-dwelling, larval phase of cestodes is usually characterized by host immunomodulatory activities [105]. The observation that some SM and CNS enriched proteins were recognized by antibodies present in the sera of infected animals, could have implications on our understanding about the modulation of the host immune response by cestode parasites.

## Conclusions

High-throughput proteomics using a TMT-multiplexed strategy allowed the identification and quantification of over 4,200 proteins across the nine samples of *T*. *solium* cysts. The *T*. *solium* cyst’s proteome constitutes a mixture of host and parasite proteins (one of each 5 proteins were of host origin). *T*. *solium* cysts obtained from either naturally or experimentally infected pigs have several proteins and antigens enriched for SM or CNS localizations. The identified host proteins were highly diverse and were involved in a number of metabolic and signaling processes. Through immuno-localization studies carried out for several host proteins, we found that they are localized in a variety of cysts tissues (tegumentary or subtegumentary tissues in the bladder wall or in the scolex). Other host proteins were detected in the vesicular fluid. Here, we also showed that several host proteins are uptaken intactly, as an example, IgG retained their antige-binding activity. Exploring the functional activity of host proteins in the cyst’s tissue certainly deserves further studies.

The parasite’s antigens that were found enriched for a certain tissue, could be used for the design of highly effective immunodiagnostic methods by combining the peptides/proteins derived from SM and CNS enriched antigens. Using several peptide mixtures and machine-learning models we were able to distinguish between cysticercotic and non cysticercotic pigs with an efficiency that is comparable to the current diagnostic methods using complex cyst’s crude extracts, however, the appropriate subset of tissue-enriched antigens remains to be identified. Development of an optimal immunodiagnostic test for human and porcine cysticercosis requires the use of SM and CNS enriched antigens; variation of those antigens in the cysts isolated from different endemic areas remains to be analyzed, though. This assessment could be crucial for the improvement of the current diagnostic tests. Characterization of the antigenic proteins enriched with each tissue localization of the cysts, is worth studying not only for the design of more efficient immunological tests for human and porcine cysticercosis, but also because they could be involved in complex tissue specific immunomodulatory processes.

## Supporting information

S1 Fig Proteomic strategy. Groups of 5 cysts each were dissected either from the skeletal muscle or the central nervous system of five cysticercotic pigs. The five cysts in each group were dissected from the same animal. Afterwards, protein extracts were obtained from each group of cysts and digested using LysC/Trypsin; the peptides were desalted, dried and labelled using TMT reagents. After labelling, the peptides were combined in a single tube and fractionated by basic pH reverse phase liquid chromatography; 12 non-adjacent pooled fractions were analyzed in an Orbitrap Fusion mass spectrometer.
(TIF)

S2 Fig Selection of antigenic peptides. More than two hundred proteins of *Taenia solium* cysts were analyzed to identify their antigenic regions. The antigenic regions were quantitatively estimated using two algorithms. Only the proteins (42) with the highest content of antigens/epitopes were furtherly screened. Only peptides that were predicted by both algorithms and subsequently analyzed using LBTOPE algorithm were considered, as a result, only one peptide was selected for each protein.
(TIF)

S3 Fig Immunohistochemistry controls. Paired section of central nervous system and skeletal muscle cysts were incubated only with the secondary antibody.
(TIF)

S4 Fig Antigenic response space using human sera. The raw optical densities were analyzed using machine-learning methodologies. A) Individual peptide testing. B) Antigenic response space using human sera, also was include a peptide named p678000 (HSTCQSCTKCPPGQGAEKPC) associated with the skeletal muscle localization of the cysts.
(TIF)

S5 Fig Sequence alignment, antigenicity and abundance of two *Taenia solium* GP50 proteins. A) Sequence alignment of the two GP50 proteins characterized here and the GP50 previously described [103]. Signal peptide sequence, GPI-anchoring site, N-glycosilation site and disulphide bond prediction are indicated on the sequence. Each GP50 was associated with a tissue localization of the cysts (B and C); D) Antigenicity and B-cell epitope predictions are shown for each protein using Kolaskar and Bepipred algorithms (see materials and methods for further details).
(TIF)

S6 Fig Cestode proteome comparisons. *Taenia solium* proteome was compared with the reported proteomes of *Echinococcus granulosus*, *E*. *multilocularis* and *Mesocestoides corti*. A) Venn diagram showing than only 14 proteins are consistently found expressed between those four parasites. B) Protein ID (referred to the *T*. *solium* genome database) and names of the 14 proteins shared between those four organisms. C) Comparison between the *T*. *solium* theoretical secretome and the proteome described here for *T*. *solium* cysts obtained from skeletal muscle and central nervous system. 187 ES proteins (out of 807 reported in the secretome were shared in both lists. D) Hypothetical protein expression; a total of 357 hypothetical proteins (without functional annotation) were identified in our study (referred to the *T*. *solium* genome database).
(TIF)

S1 Table List of antibodies used. Antibody reactivity, clonality, vendor and catalogue number of the antibodies used for western blotting and immunohistochemistry.
(PDF)

S1 Data Source code for the support vector machine. (PY)

S2 Data Proteins identified in the extracts of *Taenia solium* cysts dissected from skeletal muscle and central nervous system of infected pigs, identified and quantified by mass spectrometry. (XLSX)

S3 Data Peptides of identified in the extracts of *Taenia solium* cysts dissected from skeletal muscle and central nervous system of infected pigs, identified and quantified by mass spectrometry. (XLSX)

S4 Data Highly antigenic proteins enriched for skeletal muscle or central nervous system. (XLSX)

S5 Data Functional annotation of the tissue-enriched proteins of *Taenia solium* cysts located in the skeletal muscle and central nervous system, as well as the functional annotation of a subset of constitutive proteins and the skeletal muscle pig proteome obtained from Zhang *et al* 2015. (XLSX)

S6 Data Cestode proteome comparisons. The proteome of *Taenia solium* was compared with the reported proteomes *of Echinococcus granulosus*, *E*. *multilocularis* and *Mesocestoides corti*.
(XLSX)
