# Supplementary figures and images for: Quantitative multiplexed proteomics of Taenia solium cysts obtained from the skeletal muscle and central nervous system of pigs
Source: PLoS Negl Trop Dis. 2017 Sep 25;11(9):e0005962. doi: 10.1371/journal.pntd.0005962 (PMC5634658; doi:10.1371/journal.pntd.0005962)

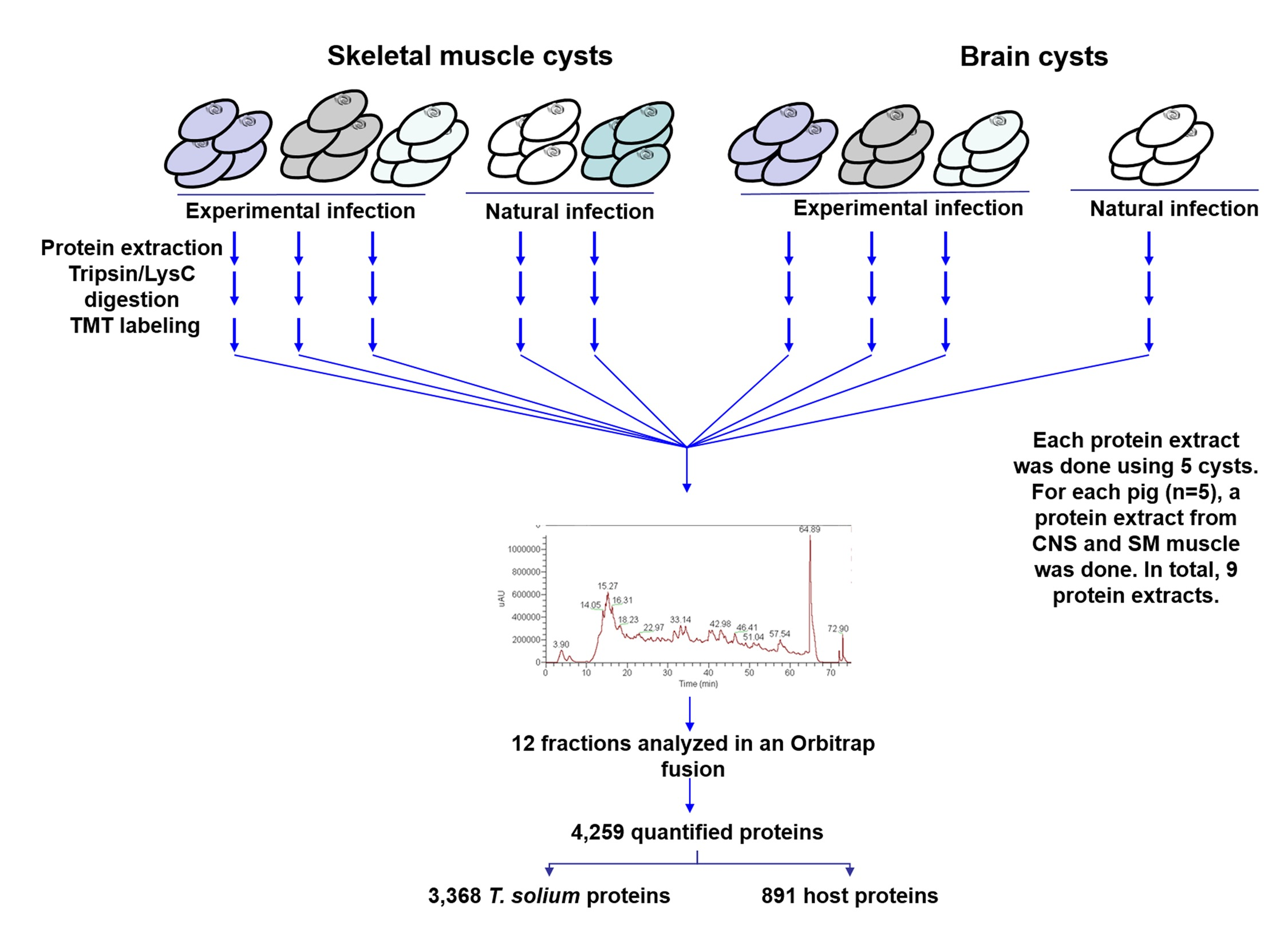

Supplement: S1 Fig — Groups of 5 cysts each were dissected either from the skeletal muscle or the central nervous system of five cysticercotic pigs. The five cysts in each group were dissected from the same animal. Afterwards, protein extracts were obtained from each group of cysts and digested using LysC/Trypsin; the peptides were desalted, dried and labelled using TMT reagents. After labelling, the peptides were combined in a single tube and fractionated by basic pH reverse phase liquid chromatography; 12 non-adjacent pooled fractions were analyzed in an Orbitrap Fusion mass spectrometer. (TIF) [file pntd.0005962.s001.tif]

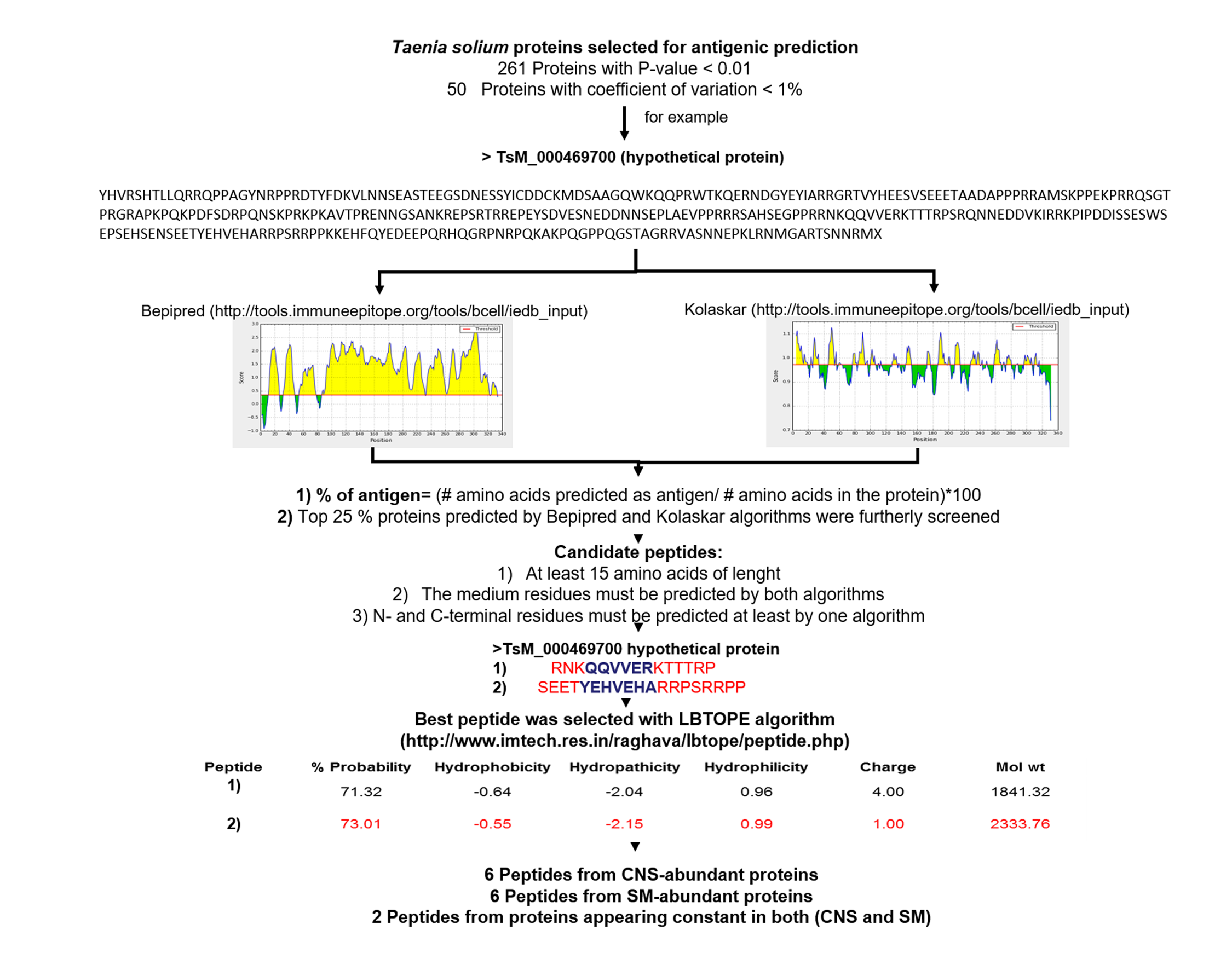

Supplement: S2 Fig — More than two hundred proteins of Taenia solium cysts were analyzed to identify their antigenic regions. The antigenic regions were quantitatively estimated using two algorithms. Only the proteins (42) with the highest content of antigens/epitopes were furtherly screened. Only peptides that were predicted by both algorithms and subsequently analyzed using LBTOPE algorithm were considered, as a result, only one peptide was selected for each protein. (TIF) [file pntd.0005962.s002.tif]

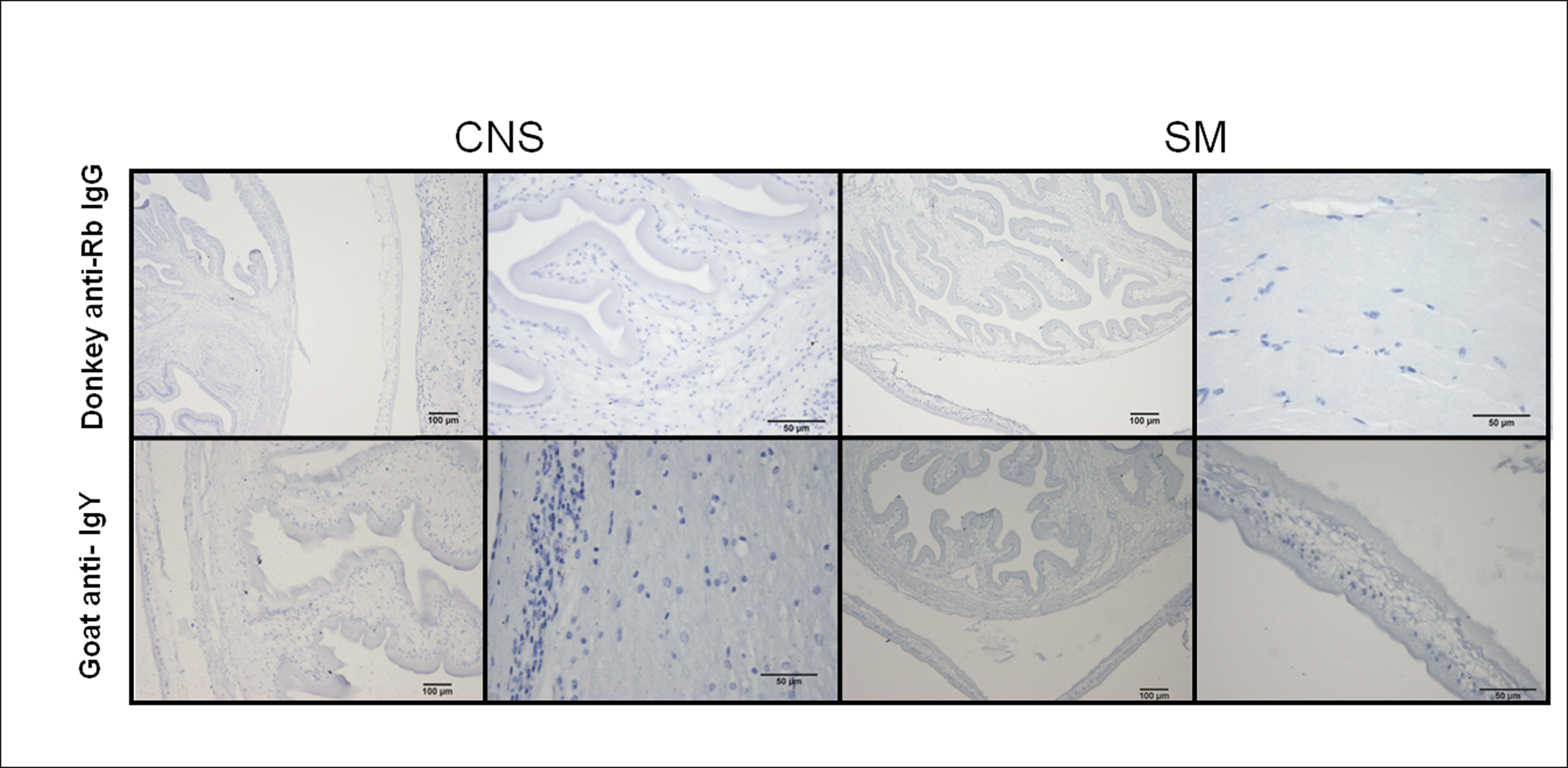

Supplement: S3 Fig — Paired section of central nervous system and skeletal muscle cysts were incubated only with the secondary antibody. (TIF) [file pntd.0005962.s003.tif]

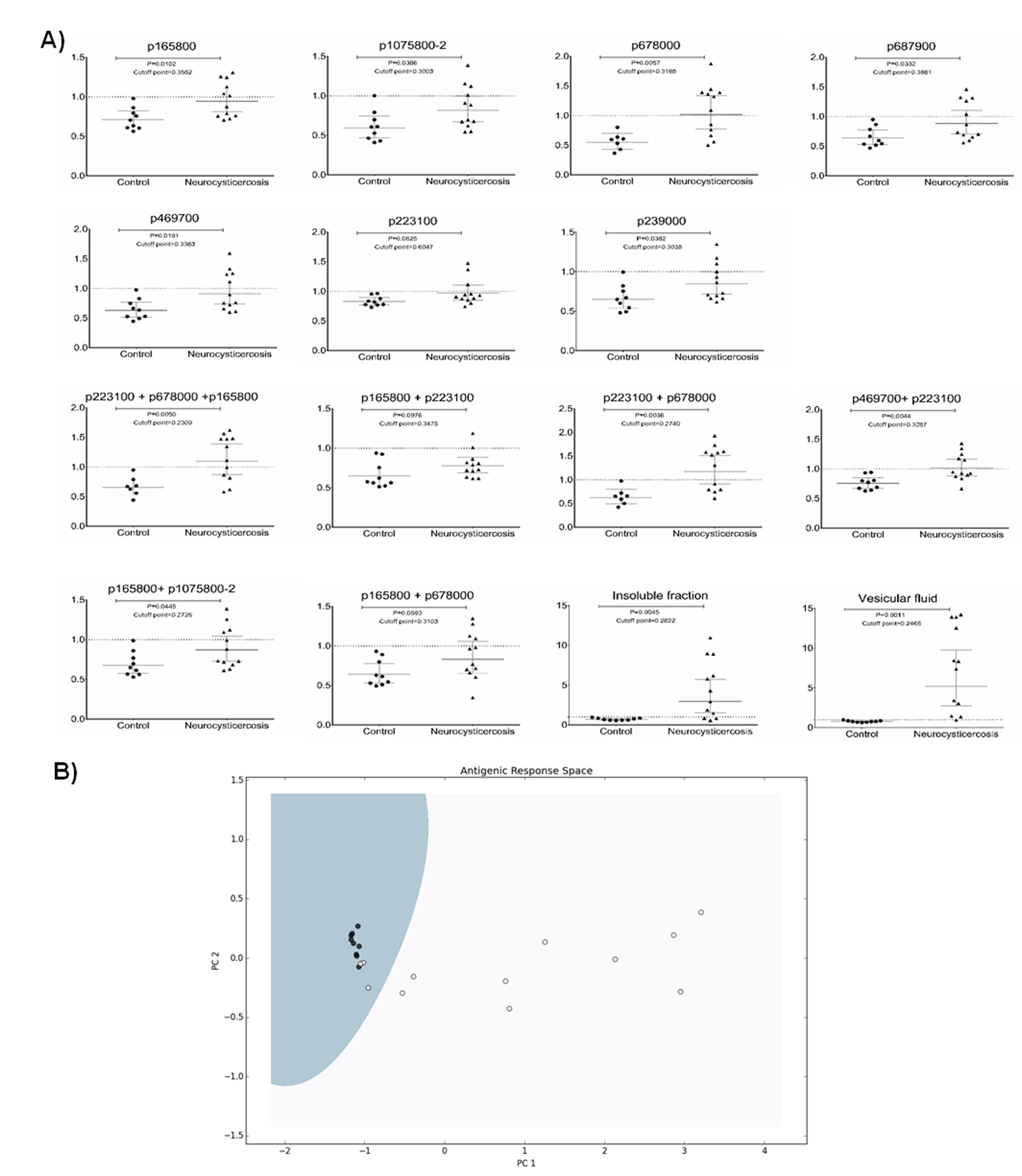

Supplement: S4 Fig — The raw optical densities were analyzed using machine-learning methodologies. A) Individual peptide testing. B) Antigenic response space using human sera, also was include a peptide named p678000 (HSTCQSCTKCPPGQGAEKPC) associated with the skeletal muscle localization of the cysts. (TIF) [file pntd.0005962.s004.tif]

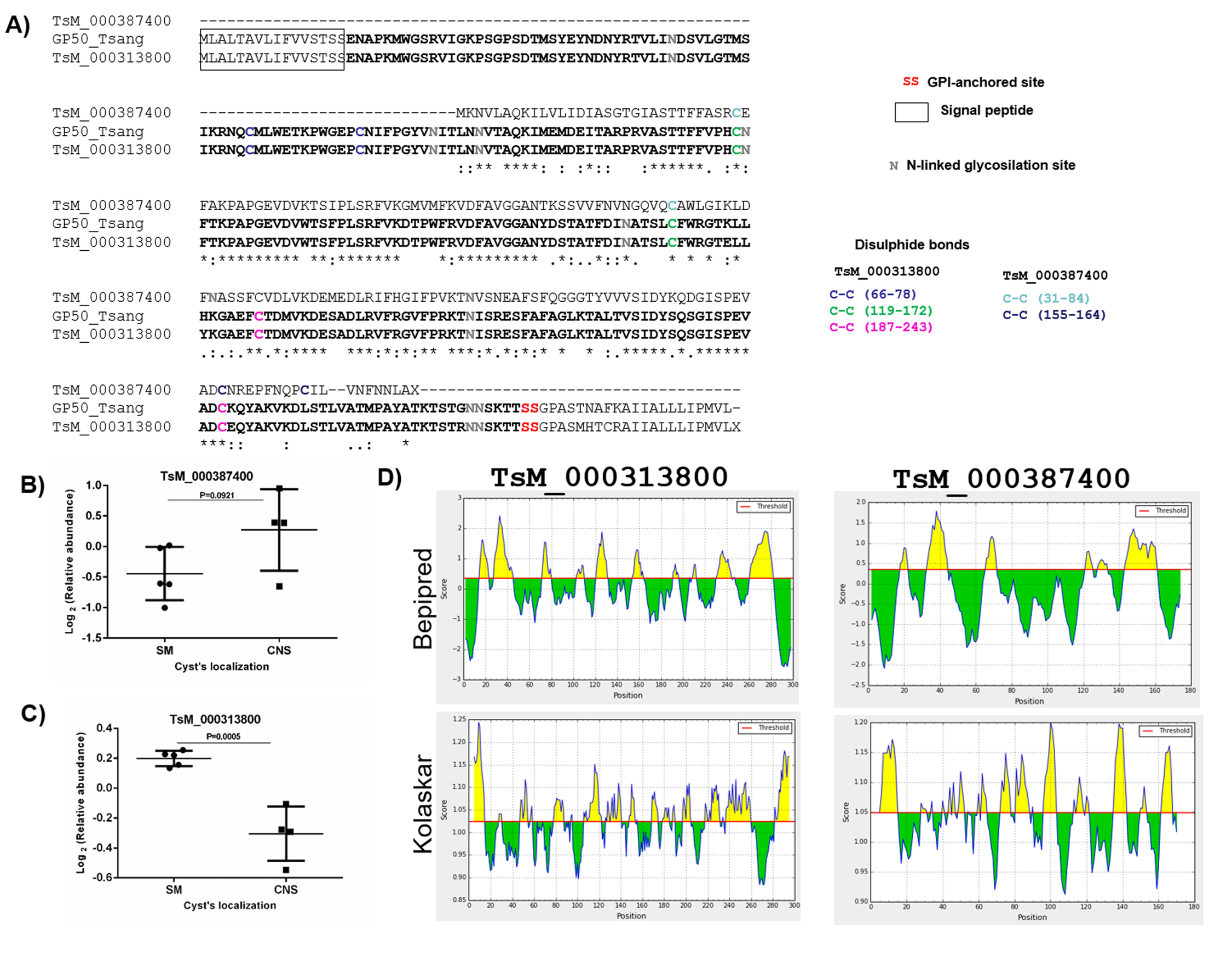

Supplement: S5 Fig — A) Sequence alignment of the two GP50 proteins characterized here and the GP50 previously described [103]. Signal peptide sequence, GPI-anchoring site, N-glycosilation site and disulphide bond prediction are indicated on the sequence. Each GP50 was associated with a tissue localization of the cysts (B and C); D) Antigenicity and B-cell epitope predictions are shown for each protein using Kolaskar and Bepipred algorithms (see materials and methods for further details). (TIF) [file pntd.0005962.s005.tif]

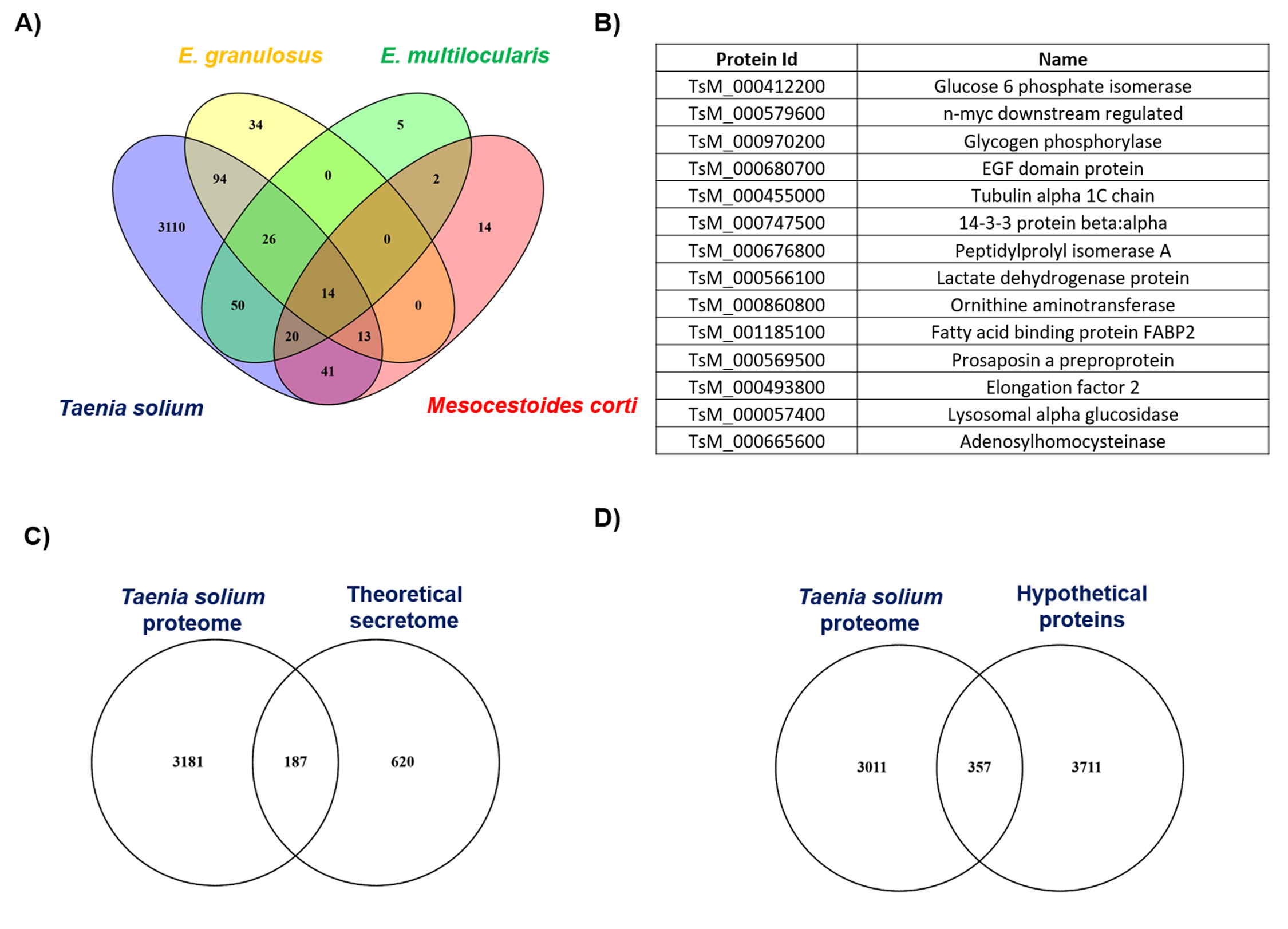

Supplement: S6 Fig — Taenia solium proteome was compared with the reported proteomes of Echinococcus granulosus, E. multilocularis and Mesocestoides corti. A) Venn diagram showing than only 14 proteins are consistently found expressed between those four parasites. B) Protein ID (referred to the T. solium genome database) and names of the 14 proteins shared between those four organisms. C) Comparison between the T. solium theoretical secretome and the proteome described here for T. solium cysts obtained from skeletal muscle and central nervous system. 187 ES proteins (out of 807 reported in the secretome were shared in both lists. D) Hypothetical protein expression; a total of 357 hypothetical proteins (without functional annotation) were identified in our study (referred to the T. solium genome database). (TIF) [file pntd.0005962.s006.tif]
