# Supplementary material for: Quantitative multiplexed proteomics of Taenia solium cysts obtained from the skeletal muscle and central nervous system of pigs
Source: PLoS Negl Trop Dis. 2017 Sep 25;11(9):e0005962. doi: 10.1371/journal.pntd.0005962 (PMC5634658; doi:10.1371/journal.pntd.0005962)
Supplement: S1 Table — Antibody reactivity, clonality, vendor and catalogue number of the antibodies used for western blotting and immunohistochemistry. (PDF) [file pntd.0005962.s007.pdf]

Name	Organism	Cat #	Isotype	Clonality	Reacts with
Anti-albumin	sheep	ab186525	IgG	polyclonal	mouse, rat, horse,rabbit, chicken, guinea pig, cow, cat, dog, human, pig
Anti-hemoglobin	goat	ab19362	IgG	polyclonal	human
Rabbit anti-sheep IgG	rabbit	ab6747	IgG	polyclonal	
Anti-haptoglobin	sheep	ab8968	IgG	polyclonal	mouse, rat, rabbit, goat,horse, cat,dog,human,pig
Anti-hemaoglobin subunit epsilon	rabbit	ab136624	IgG	polyclonal	rat
Anti-hemopexin	rabbit	ab133415	IgG	polyclonal	mouse, rat
Donkey anti-rabbit IgG	Donkey	ab6802	IgG	polyclonal	
Anti-LDL	Chicken	ab157795	IgY	polyclonal	human
Goat anti-chicken IgY	goat	ab6877	IgG	polyclonal	chicken
Anti-hepcidin-25	rabbit	ab75883	IgG	polyclonal	
Anti-ferritin light chain	rabbit	ab69090	IgG	polyclonal	mouse, rat, dog, human
